# Application of Ligand- and Structure-Based Prediction Models for the Design of Alkylhydrazide-Based HDAC3 Inhibitors as Novel Anti-Cancer Compounds

**DOI:** 10.3390/ph16070968

**Published:** 2023-07-06

**Authors:** Emre F. Bülbül, Dina Robaa, Ping Sun, Fereshteh Mahmoudi, Jelena Melesina, Matthes Zessin, Mike Schutkowski, Wolfgang Sippl

**Affiliations:** 1 Department of Medicinal Chemistry, Institute of Pharmacy, Martin-Luther University of Halle-Wittenberg, 06120 Halle (Saale), Germanydina.robaa@pharmazie.uni-halle.de (D.R.); sunpingmc@gmail.com (P.S.); jelenamelesina@gmail.com (J.M.); 2 Department of Enzymology, Institute of Biotechnology, Martin-Luther University of Halle-Wittenberg, 06120 Halle (Saale), Germanymike.schutkowski@biochemtech.uni-halle.de (M.S.)

**Keywords:** docking, binding free energy, pharmacophore, atom-based QSAR, alkylhydrazide, histone deacetylases (HDAC)

## Abstract

Histone deacetylases (HDAC) represent promising epigenetic targets for several diseases including different cancer types. The HDAC inhibitors approved to date are pan-HDAC inhibitors and most show a poor selectivity profile, side effects, and in particular hydroxamic-acid-based inhibitors lack good pharmacokinetic profiles. Therefore, the development of isoform-selective non-hydroxamic acid HDAC inhibitors is a highly regarded field in medicinal chemistry. In this study, we analyzed different ligand-based and structure-based drug design techniques to predict the binding mode and inhibitory activity of recently developed alkylhydrazide HDAC inhibitors. Alkylhydrazides have recently attracted more attention as they have shown promising effects in various cancer cell lines. In this work, pharmacophore models and atom-based quantitative structure–activity relationship (QSAR) models were generated and evaluated. The binding mode of the studied compounds was determined using molecular docking as well as molecular dynamics simulations and compared with known crystal structures. Calculated free energies of binding were also considered to generate QSAR models. The created models show a good explanation of in vitro data and were used to develop novel HDAC3 inhibitors.

## 1. Introduction

Epigenetics refers to reversible alterations in the gene expressions that do not modify the DNA sequence [1]. Post-translational modifications such as methylation, acetylation, and others introduce changes on the N-terminal tails of histones [2]. Histone acetylation and deacetylation are controlled by different classes of enzymes, namely histone acetyltransferases (HAT) and histone deacetylases (HDAC) [3,4]. Thus, chemical modification is reversible [5,6].

To date, 18 human HDACs have been characterized. HDACs are separated into two groups and four classes depending on their sequence similarity to yeast HDAC [7]. Zinc-dependent HDACs are class I, class II, and class IV HDACs, while nicotinamide adenine dinucleotide (NAD+)-dependent enzymes are class III HDACs which are also known as sirtuins [8,9,10]. Class I HDACs (HDAC1, 2, 3, and 8) are located in the nucleus [11]. HDAC1 and HDAC2 interact with the nucleosome remodeling and deacetylase complex (NuRD), transcriptional regulatory protein sin3A, corepressor of REST (CoREST), and mitotic deacetylase complex (MIDAC) [12,13,14,15,16], while HDAC3 forms a complex only with the silencing mediator for retinoid and thyroid receptors (SMRT) and nuclear receptor corepressor (NCoR) [17,18]. HDAC8 does not need to form a complex and works alone [19,20].

HDACs are involved in signal transduction, cell growth, and cell death [21]. So far, several inhibitors including SAHA, FK228, belinostat, and panobinostat have been approved by the FDA against T-cell lymphoma [22,23,24,25]. However, due to reported side effects and unfavorable pharmacokinetics, much effort has been made to develop novel selective and better bioavailable HDAC inhibitors against several diseases such as cancer, parasitic diseases, inflammation, and others [26,27,28,29].

The majority of HDAC inhibitors consist of three pharmacophore features: a zinc-binding group (ZBG), which chelates the zinc ion at the bottom of the catalytic pocket, a linker group, which is located at the lysine binding tunnel, and a cap group, which is solvent-exposed at the rim of the pocket [7,30]. Some HDACs can be selectively inhibited by compounds addressing available subpockets of HDACs such as the side-pockets, lower pocket, and foot pocket (FP) [31,32,33,34,35,36,37,38,39]. For example, class I HDACs show an additional foot pocket [40]. Targeting this foot pocket resulted in class I HDAC-selective inhibitors [31,34,35,41,42,43,44]. However, the development of selective inhibitors for the class I members of HDACs, particularly HDAC1-3, remains a critical challenge to overcome. The zinc-binding group is an integral part of most HDAC inhibitors. Until now, hydroxamic acid, 2-aminobenzamide, 2-substituted benzamide, alkyl/arylketone, and thiol groups have often been used as warheads in the inhibitors of class I HDACs [35,37,38,41,42,43,44,45].

**Figure 1:**
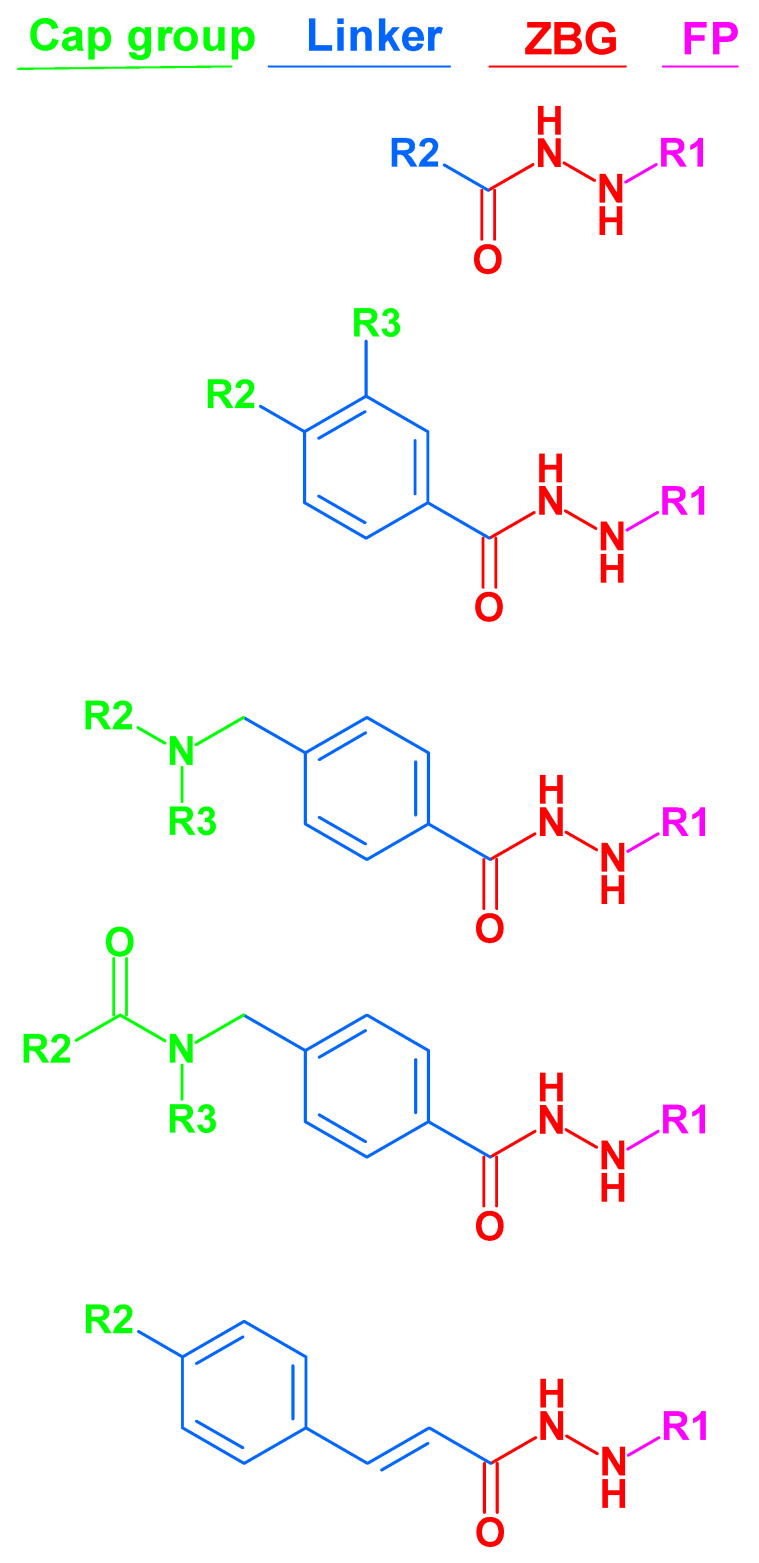
Structures of alkylated-hydrazide-based HDAC inhibitors that were used in this article. (The molecular structures of all compounds under study are shown in Table S1, Supplement). ZBG: zinc-binding group; FP: foot pocket.

Recently, Wang et al. discovered a lead compound containing a benzoylhydrazide moiety that selectively inhibits HDAC1, HDAC2, and HDAC3 [46]. These compounds showed a fast-on/slow off binding mechanism [46]. Consequently, the alkylhydrazide scaffold attracted attention for the development of HDAC3 inhibitors, and some alkylhydrazide derivatives were found to show high potency, increased selectivity, and good bioavailability [46,47,48,49,50,51,52]. Therefore, the alkylhydrazide zinc-binding group represents a promising alternative to classical hydroxamic acids. The general structures of thealkylhydrazides are shown in Figure 1.

Interestingly, increasing the length of the *N*-alkyl group (from *n*-propyl to *n*-hexyl) resulted in a shift of the selectivity towards HDAC8 and provided substrate-competitive and highly potent inhibitors [53].

HDAC3 deacetylates various histone and non-histone proteins [54]. The catalytic activity of HDAC3 is dependent on the formation of a complex with silencing of mediator co-repressor 1 (NCoR1) and retinoic acid and thyroid hormone receptor (SMRT3) [55]. As a class I HDAC member, HDAC3 deletes the acetyl mark from histone tails, resulting in a tightly packed and transcriptionally inactive chromatin structure [56]. HDAC3 has hence been implicated in several pathophysiological processes and disorders including different cancer types, inflammatory conditions such as rheumatoid arthritis, neurodegenerative disorders like Huntington’s and Alzheimer’s disease, diabetes, kidney diseases, as well as cardiovascular diseases [54,56,57,58,59,60,61,62,63]. The exact role of HDAC3 in the various pathological conditions remains poorly understood, as potent and selective HDAC3 inhibitors have been scarce. Often, the described HDAC3 inhibitors in cells are also able to inhibit the structurally very similar HDAC1 and HDAC2 [41,42,43,44,45,46]. Therefore, it is a promising task to develop effective and selective HDAC3 inhibitors.

**Figure 2:**
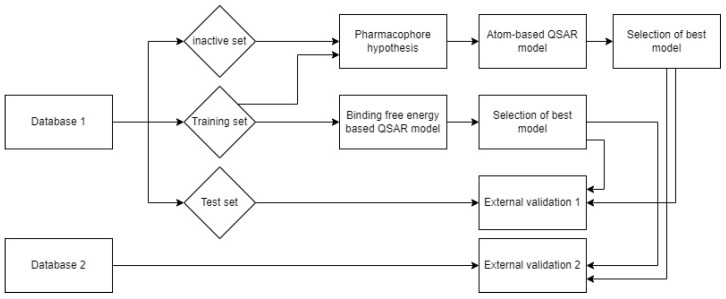
Workflow for the generation of ligand- and structure-based models for alkylhydrazide-based HDAC3 inhibitors.

In the current study, we performed docking and molecular dynamics studies of alkylhydrazides as HDAC3 inhibitors. In order to understand the structure–activity relationship of this class of inhibitors, available data were compiled to apply ligand-based and structure-based methods. Various quantitative structure–activity relationship (QSAR) methods were evaluated for this purpose, including pharmacophore models, atom-based 3D QSAR models, and binding-free-energy-based QSAR models. In addition, we tested the models on novel designed alkylhydrazides. The workflow followed in this study is shown in Figure 2.

## 2. Results and Discussion

### 2.1. Diversity Analysis of the Datasets

A dataset containing 63 compounds with an *N*-monosubstituted hydrazide scaffold was collected from the literature [47,49,51]. The 2D structures and IC_50_ values of all alkylhydrazides are shown in Table S1 (Supplement). The selected compounds cover a reasonable biological activity range (Table 1). All compounds were measured in vitro using recombinant human HDAC3 and the peptidic substrate (Boc-Lys(acetyl)-AMC). The fluorescence intensity was measured at excitation and emission wavelengths of 360 and 460 nm, respectively [47,49,51].

We first grouped the compounds into three activity classes according to their HDAC3 inhibitory data (Table 1, Table S1 Supplement):

1. Highly active inhibitors showing pIC_50_ > 7
2. Moderately active inhibitors showing pIC_50_ between 5.30 and 7
3. Inactive inhibitors showing pIC_50_ < 5.30

The compounds were randomly divided into a training set (70%; 39 compounds) and a test set (30%; 17 compounds) using the “RAND” function in the MOE program (MOE–Molecular database calculator–RAND) [64]. The compounds either having no exact IC_50_ values or showing an IC_50_ value higher than 5 µM were classified as inactive (Table 1). The same training and test sets were used for the ligand- and structure-based model development studies. The training set was used to generate the models, while the independent test set was utilized to evaluate the predictive accuracy of the selected best models. The inactive set was only utilized for the validation of the pharmacophore models by calculating the inactive-survival score (detail in Section 2.2).

**Figure 3:**
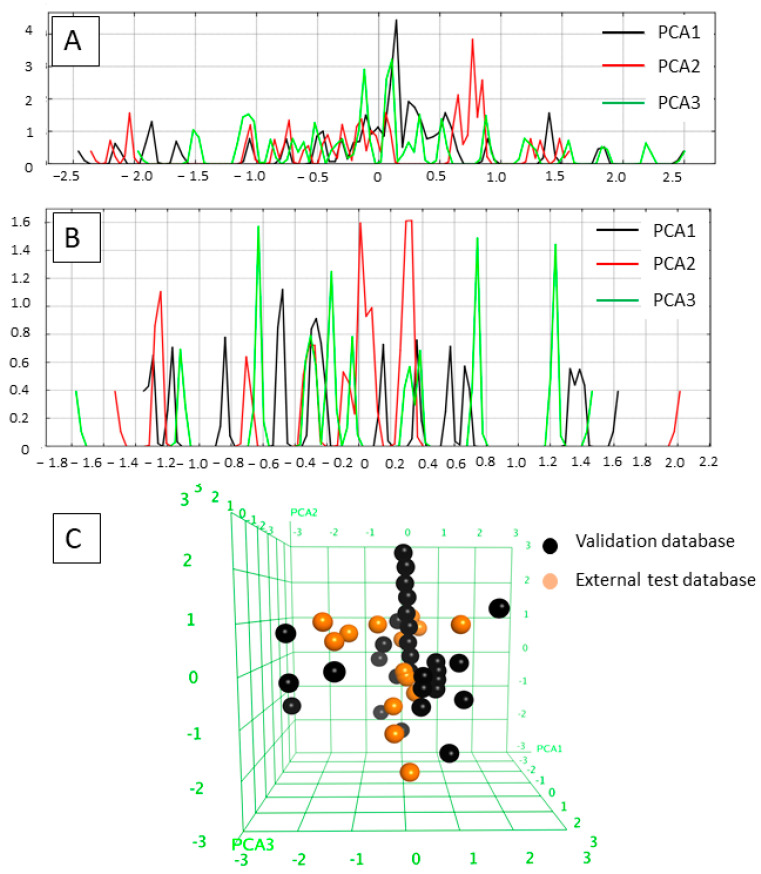
(**A**) Field histogram to visualize the variation of the three most important principal components for the training set. (**B**) Field histogram to visualize the variation of the three most important principal components for the test set. (**C**) Three-dimensional plot of the first three PCAs. The training set is colored black; the test set is colored orange.

The applicability domains of the training and external test sets were analyzed by plotting the three most important principal components (PCA1, PCA2, and PCA3) [60,65] of the calculated descriptors (PEOE_VSA_HYD, GCUT_SLOGP_0, TPSA, b_single, lip_acc, lip_don, and vsa_hyd—explained in Table 2). The most important PCA of the molecular descriptors can explain about 100% of the original space. The PCA analysis showed that the training set and external test set were homogeneously distributed in the chemical space (Figure 3).

### 2.2. Analysis of the Pharmacophore Model

An important step in establishing a 3D-QSAR model is the development of the correct alignment, usually based on a generated pharmacophore model. In the current work, the pharmacophore model was generated using the Phase module implemented in Schrödinger considering 30 active compounds (pIC_50_ > 7) [66]. Then, seven inactive compounds were used to analyze the ability of the generated models to discriminate between the active and inactive compounds.

The pharmacophore model shows the 3D (three-dimensional) structural features which might be essential for the biological activity [67,68]. Hence, the pharmacophore features that are common to the 30 active compounds showing a pIC_50_ more than 7 were investigated. In total, 38 pharmacophore hypotheses were generated and scored according to the survival score. The survival score was generated by evaluating how well the selected pharmacophore hypothesis fits to the most active inhibitors. Additionally, the Phase module penalizes the generated pharmacophore hypothesis that cannot discriminate the actives from inactives. Thus, the developed hypotheses were mapped onto the inactive compounds and scored to yield the inactive-survival score. Pharmacophore hypotheses which showed a better inactive score than survival score was discarded since it cannot discriminate between active and inactive compounds. For the selected pharmacophore hypothesis, all inactive compounds should show low fitness to the pharmacophore hypothesis.

**Figure 4:**
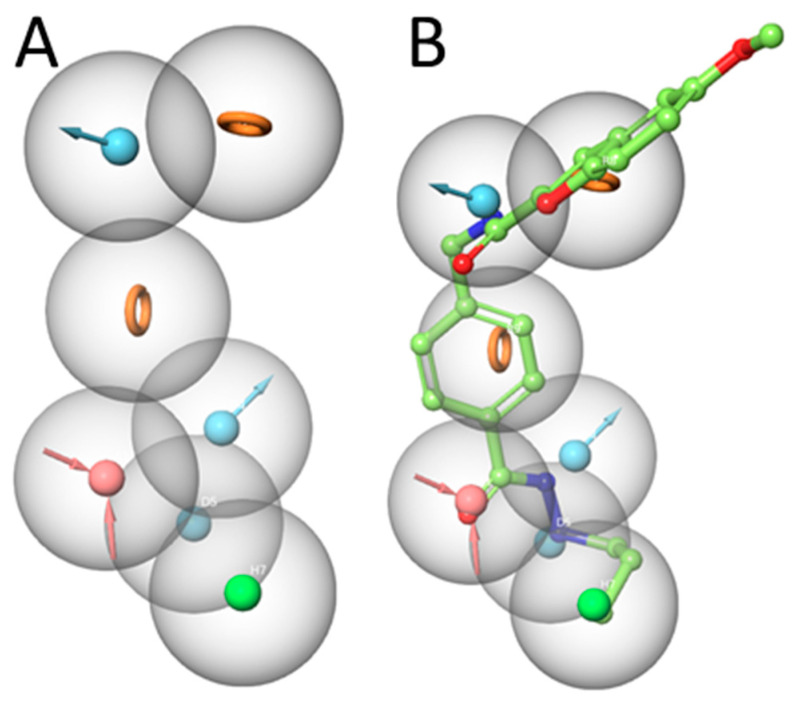
(**A**) Developed pharmacophore model (ADDDHRR) for HDAC3 inhibitors. (**B**) Superposition of compound **1** and the pharmacophore hypothesis (hydrogen bond acceptor—red color, hydrogen bond donor—cyan color, hydrophobic—green color, and ring—orange color).

After scoring the generated pharmacophore hypotheses, the best-scored pharmacophore model consisting of seven pharmacophore features (ADDDHRR–Figure 4A) was selected. The survival score (6.923) and the inactive score (1.688) of the hypothesis are shown in Table 3. The pharmacophore features were specified as the hydrogen-bond acceptor (A), the hydrogen bond donor (D), the hydrophobic (H), the negative ionic (N), the positive ionic (P), and the aromatic ring (R). It is worth noting that the less feature-based pharmacophores show weak discrimination between actives and inactives. Most inactive compounds showed a high fitness to the established pharmacophore features which led to an increase in the inactive score as shown for the DDDHRR and DDHR hypotheses (Table 3).

The generated pharmacophore model (ADDDHRR) was mapped onto the most active compound **1**. This pharmacophore model shows the importance of the hydrogen bond donor and acceptor functions of the hydrazide moiety (Figure 4B). The carbonyl oxygen of the hydrazide serves as a hydrogen bond acceptor, while the two nitrogen atoms serve as hydrogen bond donor groups. The alkyl chain shows hydrophobic features while the two aromatic ring systems are assigned as aromatic features. The amide moiety between the linker acts as a hydrogen bond donor via the amide-*NH* (details shown in the docking part). Accordingly, the selected ADDDHRR pharmacophore model shows the important structural features which can interact with the HDAC3 pocket.

In conclusion, the common pharmacophore features were determined using the active compounds in this step. The established pharmacophore model shows the required features for the binding to HDAC3. Since there is no reported X-ray structure of HDAC3 with an alkylhydrazide, the pharmacophore model gives an insight into the possible binding mode of alkylhydrazide derivatives.

### 2.3. Analysis of the Atom-Based 3D-QSAR Model

The atom-based 3D-QSAR model was built in Schrödinger19 using the 39 compounds in the training set [66,67,68]. Atom-based QSAR treats molecules as a set of overlapping van der Waals spheres. The spheres are divided into six categories: hydrogen bond donors; hydrophobic/non-polar; negative ionic; positive ionic; electron withdrawing; and miscellaneous [67,68]. The 3D-QSAR model enables us to consider all relevant structural features such as steric clashes as well as pharmacophores which play a role in the HDAC3 activity of the compounds. In this step, the previously selected seven-featured pharmacophore model (ADDDHRR) was used as an alignment rule for the generation of an atom-based QSAR model. First, 39 compounds were aligned to the pharmacophore model and then the atom-based 3D-QSAR models were generated and cross-validated. The best atom-based 3D-QSAR model was obtained with a good correlation coefficient (R^2^: 0.95) and cross-validated correlation coefficient (Q^2^: 0.88) (Table 4).

The atom-based 3D-QSAR techniques visualize the compounds as 3D (three-dimensional) based on the non-covalent protein–ligand interactions such as the hydrogen bond acceptor and donor, hydrophobic, and positive and negative ionic interactions. The model marks the favorable structural features with green cubes and unfavorable structural features with red cubes. To understand the most favorable and less favorable interactions, we analyzed all compounds from the training set. As examples, three compounds with low activity (compounds **35**, **36**, and **38**) and three compounds with good activity (compounds **1**, **2**, and **3**) from the training set were chosen to analyze the atom-based QSAR model (Figure S1, Supplement). According to the atom-based QSAR model, compound **35** exhibited poor activity due to its heptyl alkyl chain (Figure S1A, Supplement). As shown in Figure S1B (Supplement), the meta-substituent on the phenyl linker, as exemplified with compound **36**, showed an unfavorable effect on the HDAC3 activity. In the case of compound **38**, the thiophene ring showed unfavorable structural features, decreasing the HDAC3 activity (Figure S1C, Supplement). On the other hand, the propyl alkyl chain attached to the hydrazide group is favored for three active compounds (Figure S1D–F, Supplement). In addition to that, para-substituted cap groups are also favored and covered by green cubes. According to the model visualization, the least active compounds (Figure S1A–C, Supplement) are mainly covered by red cubes, while the more active compounds, especially the cap groups (Figure S1D–F, Supplement), are mostly covered by green cubes.

**Figure 5:**
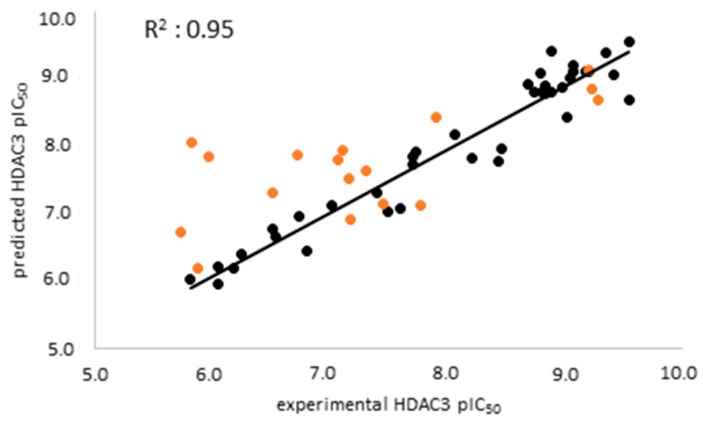
Scatter plot of the best atom-based QSAR model for the training set (black) and the test set (orange).

The external validation was performed using a test set which was not used for model generation, with the aim of evaluating the predictive accuracy and reliability of the generated atom-based QSAR model. The scatter plot of the training and test set is shown in Figure 5. The prediction results of the training, test, and inactive databases are shown in Table S2.

Analysis of the test set revealed that the atom-based QSAR model predicts the active inhibitors well, with differences less than 1 log unit. However, several of the moderately active inhibitors (compounds **51**, **53**, **55**, and **56**) in the external test set as well as the seven inactive compounds were predicted, with differences of more than 1 log unit (Table 5, Table S2 Supplement). The atom-based QSAR model classified most of the moderately active and inactive inhibitors into the active class. Due to the limited accuracy of the atom-based models in correctly predicting the inactives/weakly actives, we tried to overcome this by generating structure-based prediction models. For this, we applied the docking and binding free energy calculation techniques discussed in the next section.

### 2.4. Analyzing the Binding Mode of Alkylhydrazides in HDAC3

**Figure 6:**
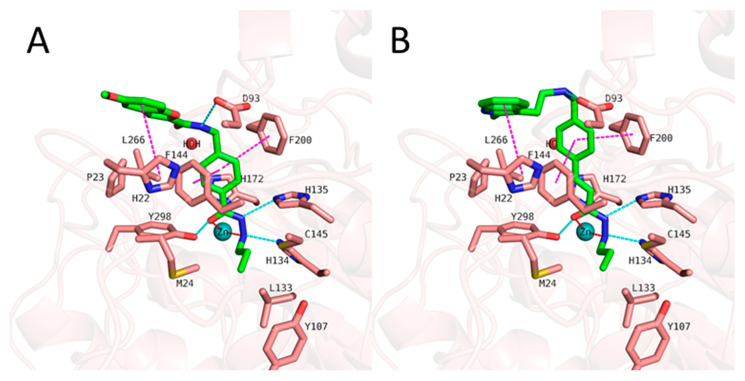
Docking poses of **1** ((**A**), green-colored sticks), **2** ((**B**), orange-colored sticks) in HDAC3 (PDB ID: 4A69). The hydrogen bonds (cyan dashed lines), hydrophobic interactions (magenta dashed lines), and metal coordination (red dashed lines) between the inhibitors and the protein are shown. Relevant residues are shown in stick representation with salmon carbon atoms in HDAC3. The zinc ion is shown as a cyan-colored sphere. The conserved water molecule is shown as a red sphere.

We started with docking all inhibitors to HDAC3 (PDB ID: 4A69 [69]) (Figure S2, Supplement). We used a docking set-up in Glide which we previously validated for HDAC inhibitors from different chemical series [31,34,53]. To date, there is no crystal structure of an HDAC in complex with an alkylhydrazide, but we have recently shown that alkylhydrazides similar to inhibitors 1 and 47 (Table S1, Supplement) [47,51] are reversible and substrate competitive inhibitors of HDACs [53]. Thus, we docked the alkylhydrazides into the catalytic pocket of HDAC3 and analyzed whether they are able to chelate the catalytic zinc ion. The analysis of the docking results of the active inhibitors, as exemplified by compounds **1** and **2** from the training set (Figure 6), showed that the hydrazide moiety chelates the zinc ion in a bidentate manner through its nitrogen and carbonyl oxygen and exhibits conserved hydrogen bond interactions with H134, H135, and Y298 at the bottom of the catalytic pocket. The aromatic linker group was placed into the hydrophobic tunnel consisting of F144, H172, F200, and L266, where it undergoes aromatic pi–pi interactions with F144 and F200. The cap group interacts with residues at the surface by forming hydrogen bond interactions with D93 as well hydrophobic interactions with H22 and P23 in HDAC3. A structural difference which influences the potency and selectivity on HDAC3 is observed in the foot pocket region. According to the docking results, the propyl and butyl chains of the alkylhydrazides fit well into the foot pocket of HDAC3. However, replacing the propyl or butyl chains by pentyl or longer side chains resulted in a dramatic decrease in HDAC3 activity due to the steric hindrance observed in HDAC3. The Y107 residue pushes L133, resulting in a narrower foot pocket region [31,34]. Hence, the pentyl and longer alkyl chains in the foot pocket region show steric clashing with M24 and L133, causing a decrease in or loss of HDAC3 activity.

Although the docking poses show reasonable binding modes in the HDAC3 active site, the correlation between the docking scores and pIC_50_ values was poor (R^2^ = 0.28 for HDAC3). Thus, we rescored the docking poses by calculating the binding free energies.

**Figure 7:**
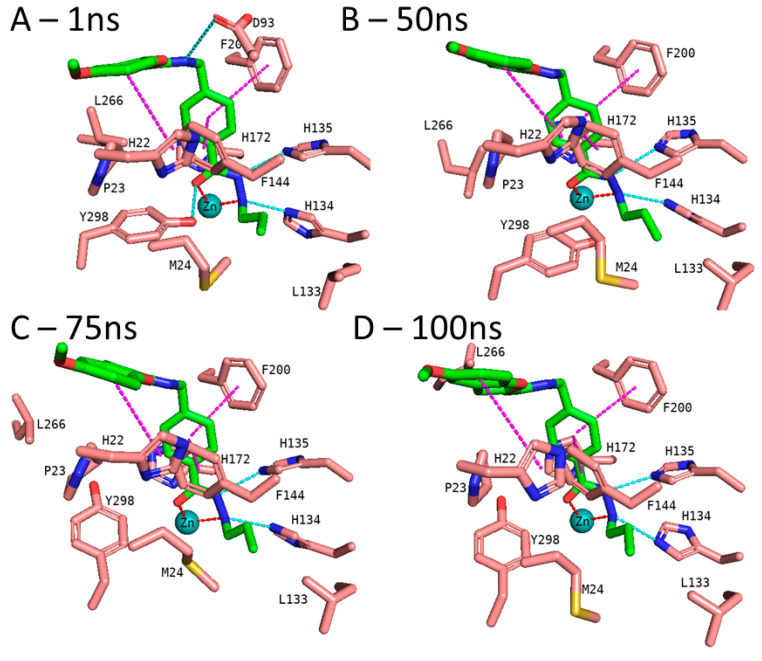
MD frames of the HDAC3-1 complex. (**A**) The frame at 1 ns MD simulation, (**B**) the frame at 50 ns MD simulation, (**C**) the frame at 75 ns MD simulation, and (**D**) the frame at 100 ns MD simulation. The hydrogen bonds (cyan dashed lines), hydrophobic interactions (magenta dashed lines), and metal coordination (red dashed lines) between the inhibitors and the protein are shown. Relevant residues are shown in stick representation with salmon carbon atoms in HDAC3. The ligand is shown in stick representation with green carbon atoms. The zinc ion is shown as a cyan-colored sphere.

**Figure 8:**
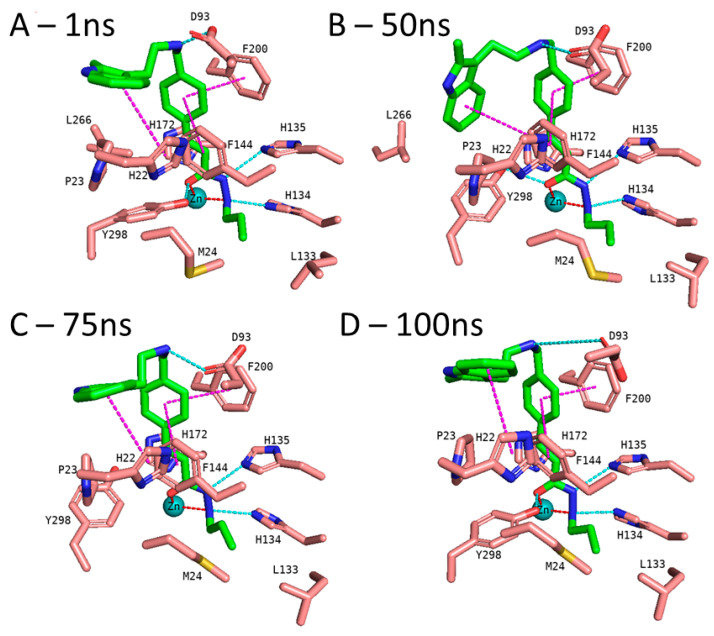
MD frames of the HDAC3-2 complex. (**A**) The frame at 1 ns MD simulation, (**B**) the frame at 50 ns MD simulation, (**C**) the frame at 75 ns MD simulation, and (**D**) the frame at 100 ns MD simulation. The hydrogen bonds (cyan dashed lines), hydrophobic interactions (magenta dashed lines), and metal coordination (red dashed lines) between the inhibitors and the protein are shown. Relevant residues are shown in stick representation with salmon carbon atoms in HDAC3. The ligand is shown in stick representation with green carbon atoms. The zinc ion is shown as a cyan-colored sphere.

In addition to the docking results, we checked the stability of the predicted interactions of the potent inhibitors 1 and 2 with the binding site using 100 ns MD simulation (Figure 7, Figure 8, Figures S3 and S4, Supplement). MD simulation analysis of compounds **1** and **2** revealed that the *n*-propyl chain attached to the hydrazide fit into the foot pocket consisting of M24 and L133. Notably, M24 and L133 play a key role as a gate keeper in the foot pocket region of HDAC3. M24 and L133 closed the foot pocket and made the volume narrower where only a propyl or butyl side chain favorably fit. This conformational change of M24 and L133 might explain the decrease in or loss of HDAC3 activity of the compounds with longer alkyl chains than butyl and propyl. The zinc-binding group which is the common part of compounds **1** and **2** preserves its bidentate coordination and undergoes hydrogen bond interactions with H134, H135, and Y298 throughout the MD simulation. Furthermore, the linker groups of compounds **1** and **2** remain sandwiched between F144 and F200. Besides these similar protein–ligand interactions of compounds **1** and **2**, the MD simulation analysis displayed some differences in the cap region of compounds **1** and **2**.

According to the MD simulation of compound **1**, the selected docking pose was stable during the 100 ns MD simulation (Figure 7 and Figure S3, Supplement). Throughout the MD simulation, the ligand maintained the predicted binding conformation, albeit two of the predicted interactions were lost, namely the hydrogen bond interaction between the amide group and D93 as well as the interaction between the hydrazide-carbonyl-O and Y298, due to the flexibility of the latter residue (Figure 7). The hydrogen bond distances of the HDAC3-inhibitor 1 complex throughout the 100 ns MD simulations were analyzed and plotted in Figure S4 (Supplement). No significant fluctuation was observed for the benzofuran cap group of compound **1**, which remains embedded in a hydrophobic pocket and undergoes aromatic interaction with H22 at the surface of the protein.

In the case of compound **2** (Figure 8), the flexible 2-methylindole cap group showed conformational changes. Hence, the ligand RMSD of compound **2** showed higher fluctuations (Figure S5, Supplement). During the MD simulation, the 2-methylindole group showed two different orientations: between 40–60 ns of the MD simulation, the cap group adopts an orientation where it undergoes edge-to-face interaction with F144. For the rest of the simulation time, the cap group showed the initially observed position and interacted with H22. In contrast to compound **1**, Y298 showed less fluctuation and maintained its interaction with compound **2**. Throughout the 100 ns MD simulation, compound **2** showed stable binding and maintained its bidentate chelation with the zinc ion. The hydrogen bond distances of the HDAC3-inhibitor 2 complex throughout the 100 ns MD simulations were analyzed and plotted in Figure S6 (Supplement).

In conclusion, since so far no X-ray structure has been released of an HDAC with an alkylhydrazide inhibitor complex, we docked the compounds to HDAC3 to examine the putative binding mode and to rationalize the observed SAR. Additionally, the observed proteinligand interactions were analyzed by MD simulations. The interaction at the catalytic pocket was found to be highly stable whereas some fluctuation was observed for the flexible capping groups that are located at the solvent-exposed part of the binding pocket. To provide further support for the predicted binding poses of the alkyl hydrazides, we previously examined the substrate competition of two alkyl hydrazides for the related class I member HDAC8 and confirmed that they reversibly inhibit and exhibit competitive substrate binding. However, cocrystal structures of HDACs with alkylhydrazide-based inhibitors have to be obtained to confirm the modeling results.

### 2.5. Binding Free Energy Calculation

**Figure 9:**
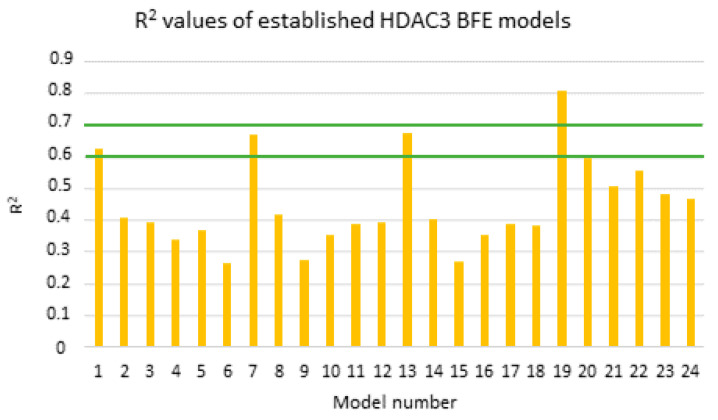
Calculated R^2^ values of the established models for alkylhydrazides (training set: orange bars). Green lines highlight the 0.6 and 0.7 R^2^ values.

Due to the low correlation between the docking scores and pIC_50_ values, rescoring of the selected docking poses was performed using the MM/GBSA method in AMBER16 [70]. The total energies of HDAC3–inhibitor complexes were calculated using four different parameter settings (solvation models) and six different frame settings (see the Methods part for details). The same training set including the 39 compounds that was used for the atom-based 3D-QSAR model was also used for model generation based on the calculated binding free energies of the compounds. In total, 24 models were generated. The models were assessed based on the correlation coefficients (R^2^) between the biological data and the calculated energy values, taking into account Tropsha’s criteria for reliable QSAR models [71] (Figure 9). The prediction results of the training, test set, and inactive compounds are shown in Table S3 (Supplement).

According to the Tropsha criteria [71], a good QSAR model should abide by the following rules; R^2^ > 0.6 and Q^2^ > 0.5. Based on the mentioned rule, models showing a calculated R^2^ value > 0.6 were considered for further statistical analysis (MODEL1, 7, 13, and 19). Interestingly, the four selected models are all based on the protein–ligand complex obtained with one minimization step (Emin1, Table 6). Further internal validation of the selected models was analyzed using the leave-one-out (LOO) method, 3-fold-cross-validation (cv), and 10-fold cv.

**Figure 10:**
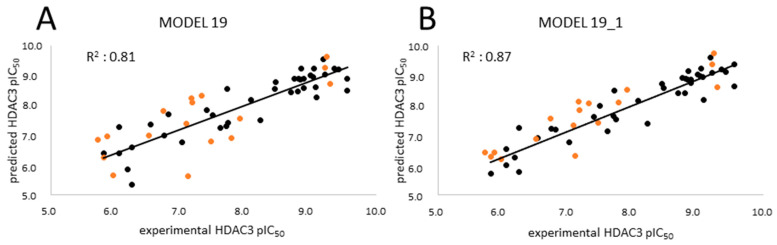
Correlation plots of (**A**) MODEL19 and (**B**) MODEL19_1 showing correlations between the predicted data and experimental data for HDAC3. Training set (black); external test set (orange).

The four selected models showed R^2^ > 0.6 and Q^2^ > 0.5. Among the selected four models, the GB8 (GBNeck) implicit solvation model outperformed the other methods (GB^HCT^ refers to GB1, and GB^OBC^ refers to GB2-5 in the article). The reason might be that the GBNeck model (referred to GB8) was generated to correct the van der Waals surface that is inaccessible to water [72]. This improvement in GB8 might help to obtain better results for the compounds used in this article. Model 19 based on the GB8 implicit solvation model showed the highest R^2^ and Q^2^ values (LOO-method) with 0.81 and 0.78, respectively, and the lowest RMSE and QMSE values with 0.49 and 0.52, respectively (Figure 10 and Table 6). In addition, we tested whether the inclusion of a 2D descriptor for the shape/electronic properties of the inhibitors could improve the models. Two-dimensional descriptors were computed for all compounds in MOE [64]. All available 2D descriptors were then assessed for their ability to improve the model. The total hydrophobic van der Waals surface area (PEOE_VSA_HYD) gave the best improvement. The combination of this 2D descriptor and energy term improved the R^2^ from 0.81 to 0.87 and the Q^2^ (LOO) from 0.78 to 0.84. In addition, this model (MODEL19_1) exhibited lower RMSE and QMSE values compared to MODEL19 (Table 6).

The test set was used to evaluate the accuracy of the best generated model (Model19_1). In the test set (Table 7), all compounds were predicted with less than 1 log unit difference. Additionally, the prediction of the inactive compounds was more satisfying compared to the previously described atom-based QSAR models, with a difference of less than 1 log unit except for compound **61** (Table S3, Supplement). For compound **61**, the docking poses could not explain the incorrect prediction. The scatter plot and prediction results are shown in Figure 10 and Table 7 and Table S3 (Supplement), respectively.

### 2.6. Evaluation of the Generated Models on Newly Designed Compounds

**Figure 11:**
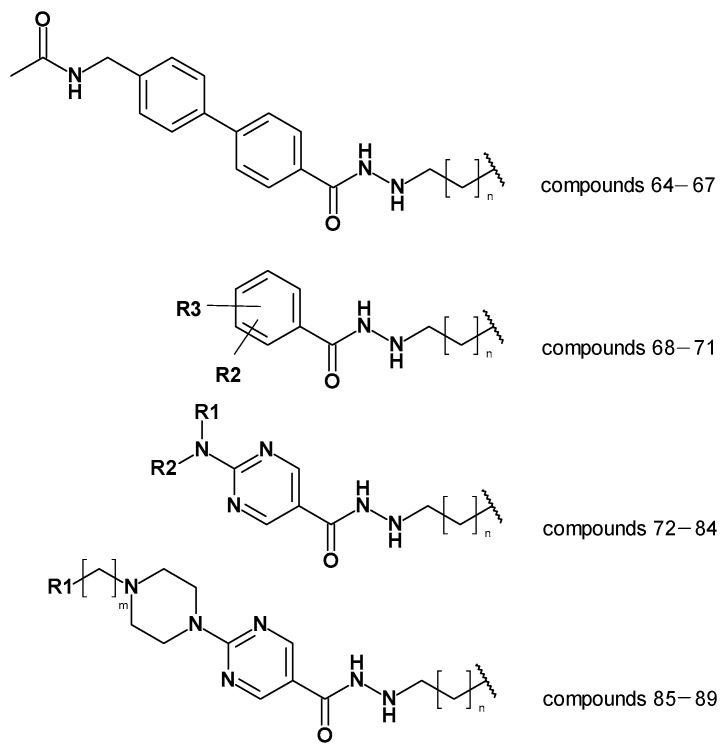
General structures of the newly synthesized compounds [53].

The created models, the atom-based 3D QSAR model and Model 19_1, and the generated docking poses were then used to predict alkyl hydrazides with other linkers and capping groups that were synthesized (chemistry and in vitro testing were published elsewhere [53]). These compounds were designed starting from compound **47** (Table S1, Supplement), where several structural modifications were introduced to extend the SAR on this series of HDAC3 inhibitors. In the first series of compounds, the effect of a different length for the alkyl side chain was evaluated. In the second series of compounds, different substitutions on position 3 or 4 of the phenyl ring were introduced. In the next series of compounds, the aminopyrimidine linker group with different *N*-arylmethyl, *N*-arylethyl, or *N*-ethylpiperazinyl moieties were tested, while in the last series of compounds, piperazinyl-piperidine linker groups were attached to the phenylalkylhydrazide core of the compounds. The general structures of the new inhibitors are summarized in Figure 11. The experimentally determined HDAC3 IC_50_ values and the prediction results are shown in Table 8, Tables S5 and S6 (Supplement, all 2D structures are summarized in Table S4, Supplement).

First, the ligand-based models; i.e., the pharmacophore models and 3D_atom-based QSAR models, were tested. The developed pharmacophore model was used to align the 26 new compounds to apply the atom-based QSAR model.

Analyzing the atom-based prediction results of the 26 new compounds revealed interesting results (Table 8). Based on the atom-based QSAR model prediction results, the absolute difference between the experimental and predicted pIC_50_ of the nine compounds, which are moderately active or inactive, was more than >1 log unit, i.e., these compounds were predicted to be more active than experimentally determined. The atom-based QSAR model makes predictions based on the effect of electron-withdrawing groups, electron-donating groups, and hydrophobic groups of compounds considering the created pharmacophore hypothesis. However, in the case of HDAC3, the shape of the foot pocket plays an important role in the inhibitor activity. Due to the smaller volume of the HDAC3 foot pocket, compounds with alkyl groups longer than butyl are moderately active or inactive as determined by the in vitro results. Atom-based QSAR models do not take the pocket volume into account, hence resulting in the observed weak prediction of these derivatives. In conclusion, the atom-based QSAR model showed a weak discriminatory power.

**Figure 12:**
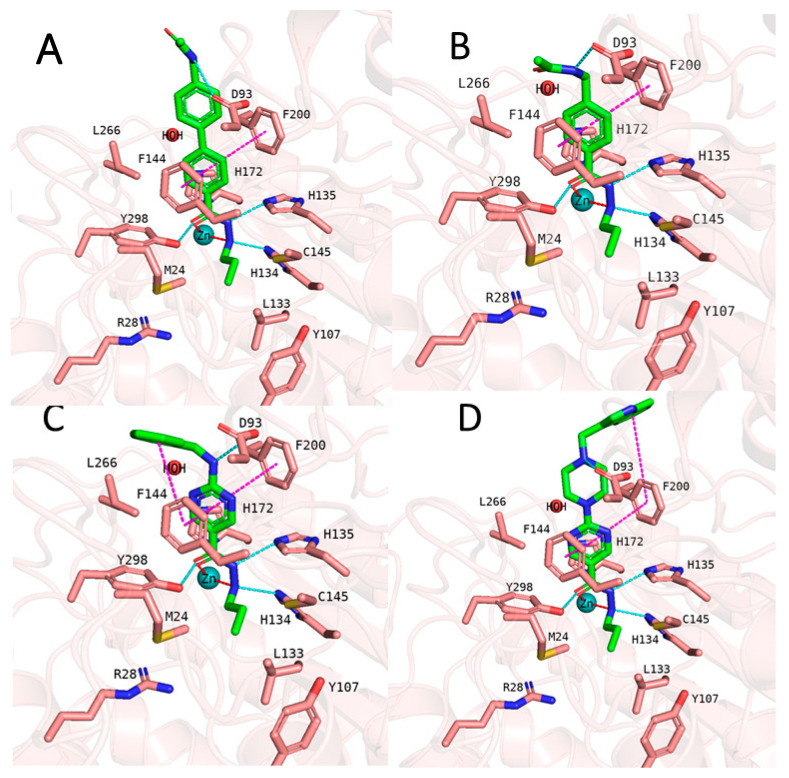
(**A**) Docking pose of compound **64**, (**B**) docking pose of compound **68**, (**C**) docking pose of compound **72**, and (**D**) docking pose of compound **85**. The hydrogen bonds (cyan dashed lines), hydrophobic interactions (magenta dashed lines), and metal coordination (red dashed lines) between the inhibitors and the protein are shown. Relevant residues are shown in stick representation with salmon carbon atoms in HDAC3. The ligand is shown in stick representation with green carbon atoms. The zinc ion is shown as a cyan-colored sphere.

Then we evaluated the binding-free-energy-based prediction results (Table 8). Initially, all 26 compounds were docked to HDAC3 using the same protocol as for the training set. Similar docking poses in HDAC3 were obtained for all 26 new compounds as obtained for compounds **1** and **2** (exemplified in Figure 12). The docking studies showed that the hydrazide moiety as well as the aromatic linker groups of all 26 compounds exhibited similar interactions as observed for compounds **1** and **2**. A bidentate chelation between the zinc ion and hydrazide moiety was observed for all compounds. In addition, the hydrazide moiety showed hydrogen bonds with H134, H135, and Y298 in the zinc-binding region of the HDAC3 catalytic pocket. The aromatic linker groups were accommodated into the hydrophobic tunnel and interacted with F144 and F200, showing pi–pi interactions. Meanwhile, the cap groups and foot-pocket-targeting groups showed significant differences which has an impact on the HDAC3 activity.

In the first series, exemplified by compound **64** (Figure 12), the acetoamidomethyl cap group was placed at the entrance of the pocket and showed hydrogen bond interactions with D93. The different length of the hydrazide alkyl side chain resulted in a significant difference in HDAC3 activity. Compound **64** possessing a propyl side chain was predicted to be more active than **65**, **66**, and **67**, which is in line with the experimentally determined data. The difference between the experimental and predicted values is indeed less than <1 log unit for this series. Moreover, compound **67** with a hexyl side chain was predicted to be inactive. This result confirms that the BFE model is sensible to the side chain effects on this dataset.

In the second series, only compound **68** (Figure 12) bearing the acetamidomethyl cap group and propyl side chain in the foot-pocket-targeting region showed moderate activity on HDAC3. The other compounds **69**, **70**, and **71** with a hexyl side chain, did not show significant activity on HDAC3. Similar to the experimentally determined activity, the BFE model also predicted compound **68** as a moderate inhibitor, while **69**, **70**, and **71** were predicted as inactive compounds.

In the third series of compounds bearing an aminopyrimidine moiety as a linker group, different *N*-arylmethyl, *N*-arylethyl, or *N*-methylpiperaziniyl moieties as capping groups were tested. This series is exemplified by compound **72** (Figure 12). All compounds bearing a propyl side chain except compound **74** were predicted with less than <1 log unit compared to the experimentally determined activities. Interestingly, compound **74** bearing an *N*-arylethyl cap group did not show significant inhibitory activity (41%@1µM) on HDAC3; however, it was predicted by the model as an active inhibitor. The flexible cap group could be the reason for the reduced activity on HDAC3. Finally, compounds **81**–**84** possessing a hexyl side chain were predicted as inactive inhibitors which is in line with the experimental findings.

The last series of compounds bearing indole or *N*-methylindole groups attached via a methyl or ethyl linker to the piperazinyl–pyrimidine scaffold were predicted close to the experimental activities. The difference between the experimental and predicted values is less than <1 log unit. The docking poses of this series are exemplified by compound **85** (Figure 12).

In conclusion we applied the validated BFE models to the further development of the alkylhydrazide-based class I HDAC inhibitors. The best inhibitors from this series were also tested for their immunmodulatory effects in Jurkat cells and showed promising cellular effects [53]. As we have recently demonstrated a potent T cell memory response by combined class I HDAC inhibition and immune-checkpoint blockade in hepatocellular carcinoma (HCC) therapy, the new alkylhydrazides represent an interesting class of inhibitors to explore their potential for cancer therapy.

## 3. Materials and Methods

### 3.1. Ligand Database Preparation

A ligand dataset of 63 compounds with hydrazide as the zinc-binding group (ZBG) was collected from the literature [47,49,51]. Only compounds having an *N*-monosubstituted hydrazide scaffold were considered. The IC_50_ values of the selected compounds were retrieved from three publications and they all were determined against HDAC3 using the same fluorogenic substrate (Boc-Lys(acetyl)-AMC (amino methyl coumarin)). The same human recombinant HDAC3 enzyme was used for the in vitro studies [47,49,51]. The compounds were prepared in ligprep tool using the OPLS3e forcefield in Schrödinger suite [73]. Subsequently, the output of the ligprep step was submitted the Confgen to generate 64 conformers per ligand while minimizing the output conformers using the OPLS3e forcefield [73,74]. The compounds were automatically divided into a training (70%) and external test set (30%) using the “RAND” function in the MOE program (MOE–Molecular database calculator–RAND) [64]. The same training set and external test set were used for the model development studies. The compounds with no exact IC_50_ values were considered as inactive. The QSAR models were built using the most active and moderate inhibitors for which exact IC_50_ values were available.

The diversity analysis of the compounds was performed by analyzing the three most important principal components using the principal component analysis (PCA) implemented in MOE [60,64,65]. The 2D descriptors were computed in MOE [64]. Several 2D descriptors were selected using the Contingency tool in MOE. The three most important principal components (PCA1, PCA2, and PCA3) were calculated using the selected 2D descriptors. These principal components were used to check the diversity of the compounds.

The 26 compounds were collected from the article published by our group to evaluate the established models and check their reliability in different datasets [53]. The ligands were prepared using the same protocol as used for the validation set.

### 3.2. Pharmacophore Model

The pharmacophore model was established using 30 inhibitors with IC_50_ values lower than 100 nM in the training set and the 7 inactive compounds in the Phase module of Schrödinger [66]. The compounds were prepared in ligprep using the OPLS3e forcefield in the previous step [73,74]. The conformational search was performed in the Phase module by adjusting 64 conformers per compound and minimizing the output conformers using the “Develop Pharmacophore model” module in Schrödinger [66]. The common pharmacophore hypotheses were developed, scored, and ranked. The selected pharmacophore model was used as an alignment rule for the atom-based 3D-QSAR model.

### 3.3. Atom-Based 3D-QSAR Model

The ligand-based 3D-QSAR model was generated using the training dataset in the Phase module of Schrödinger [66]. The 39 compounds in the training database were aligned using the selected pharmacophore hypothesis from the previous step. The QSAR models were built with four latent factors and 1.0 Å grid spacing as well as the leave-one-out-cross-validation approach. The generated models were evaluated by means of standard deviation of the regression (SD), R^2^ (correlation coefficient of regression), RMSE (root mean square error of test set prediction), and Q^2^ (cross-training of test set prediction).

### 3.4. Docking Study

The hydroxamic acid scaffold and hydrazide scaffold are structurally similar groups. Therefore, the X-ray crystal structures of HDAC2 (PDB ID: 4LXZ [35]) and HDAC3 (PDB ID: 4A69 [69]) were retrieved from the Protein Data Bank (PDB, rcsb.org [(accessed on 20 May 2022) [75]) and analyzed in MOE [64]. SAHA with a hydroxamic acid scaffold in complex with the HDAC2 protein (PDB ID: 4LXZ) was defined as a pan-HDAC inhibitor and showed activity on HDAC3 [35]. First, HDAC2 (PDB ID: 4LXZ) and HDAC3 (PDB ID: 4A69) were superposed in MOE [64]. Then, SAHA was transferred from the HDAC2 protein (PDB ID: 4LXZ) to HDAC3 to mimic the induced fit effect of the zinc-binding group.

The HDAC3–SAHA complex was prepared in the protein preparation wizard of Schrödinger’s suite by adding hydrogen bonds and missing side chains and assigning the bond orders [73]. The water molecules (except W2083) and ions (except Zn^+2^ ions) were deleted. The protonation states and tautomers were optimized at pH 7.4 using the PROPKA tool. The optimized complex was minimized using the OPLS3e force field to remove the steric clashes [74].

Molecular docking studies were carried out by applying the standard precision (SP) mode in Glide implemented in Schrödinger Suite [73]. The grid box including the information on the active site coordinates of the proteins was defined with a 10 Å radius around the ligand. Ten docking poses were employed for further post-docking minimization. The other settings were kept as the default. The docking results were visually analyzed in the MOE program [64].

### 3.5. Molecular Dynamics Simulation

The selected docking poses of compounds **1** and **2** in complex with HDAC3 (PDB ID: 4A69) were subjected to a 100 ns MD simulation in AMBER16 [70]. The Antechamber package was used to prepare the topologies, force field parameters, atom types, and bond types by applying the semi-empirical Austin Model1 with bond charge correction (AM1-BCC) [76,77]. Then, the tLEaP module was employed to prepare the protein–ligand complexes. General amber force field (GAFF), the Duan force field (ff03.r1), and 12-6-4LJ ionic model were used for the ligand, protein, and zinc, respectively [78,79,80,81]. The system was solvated by the TIP3P water model and a margin of 10 Å. Two minimization steps including the two sub-steps in each minimization were carried out. In the first step, 4000 iterations (2000 cycles of steepest descent and then 2000 of the conjugate gradient) were performed, while the protein residues, ligand, and zinc ion were restrained to their initial geometries (force constant of 10 kcal*mol^−1^* Å^−2^) to relieve the bad contacts. In the second step, 4000 iterations (2000 cycles of steepest descent and then 2000 of the conjugate gradient) were performed to remove the steric clashes in the entire complex. The restraint on the protein, ligand, and zinc were removed during the second minimization. Then, the system was heated at 300 K through 100 ps of MD. The protein–ligand complex was restrained to prevent large structural deviations (force constant of 10 kcal*mol^−1^* Å^−2^). The SHAKE algorithm was activated to constrain bonds involving hydrogens [82]. Finally, the system was equilibrated within a period of 200 ps. Langevin dynamics was applied to keep the temperature at 300 K with a collision frequency of 2 ps [83]. The pressure was kept at 1 bar using isotropic position scaling with a relaxation time of 2 ps. Afterwards, a 100 ns MD simulation was run with a time step of 2 fs using the same conditions as in the equilibration step. A non-bonded cut-off distance of 10 Å was used. The electrostatic interactions were calculated by applying the particle mesh Ewald (PME) method. After the MD simulation, CPPTRAJ module of AMBER was used to analyze the MD snapshots.

### 3.6. Binding Free Energy Calculation

The binding free energies (BFE) of the prepared protein–ligand complexes were calculated using the AMBER16 program [70]. The MMPBSA.py script was utilized for the calculations [84]. Different implicit solvent models (GB ^HCT^ (igb = 1), GB ^OBC^ (igb = 2), GB ^OBC2^ (igb = 5), and GBn (igb = 8)) were tested [85,86,87]. Molecular mechanics (MM) and solvent models were combined for the MMGBSA calculations [88,89,90]. Short 2 ns MD simulation was performed for all BFE calculations. The results of BFE were analyzed using the following six different methods: (1) a single frame at the first minimization step (Emin1), (2) a single frame at the second minimization step (Emin2), (3) a single frame at the third minimization after MD (Emin3), (4) 1–50 frames during MD (MD-1) with an interval of 5, (5) 51–100 frames during MD (MD-2) with an interval of 5, and (6) 101–500 frames during MD (MD-3) with an interval of 5. The correlation between biological activity and the energy results was measured by using the QSAR tool in the MOE program [64].

## 4. Conclusions

In the current study, we have evaluated several QSAR models including ligand-based and structure-based techniques to understand the structure–activity relationship of alkylhydrazides developed as HDAC3 inhibitors. Additionally, the binding modes of the two most potent HDAC3 inhibitors (compounds **1** and **2**) were verified through 100 ns MD simulation since there is no X-ray structure crystallized with an alkylhydrazide derivative. With the aid of ligand-based and structure-based approaches, in-house computational models have been developed for the prediction of the HDAC3 inhibitory activity of the alkylhydrazide scaffold.

The ligand-based models enabled us to obtain a general overview of the binding of the compounds in the HDAC3 protein. The established pharmacophore model and atom-based 3D-QSAR model can be used to filter the big databases. Since the shape of the foot pocket of HDAC3 has a crucial impact on the HDAC3 inhibitory activity, the predictive power of the ligand-based models was not satisfactory. These models predicted moderate inhibitors and inactive compounds as active compounds, although they predicted the actives as actives. Thus, these methods should be used to reduce the number of compounds in the big database.

The weakness of the ligand-based methods directed us to generate the structure-based methods. The binding mode of the alkylhydrazide was predicted by docking. The selected binding modes from the validation set (compounds **1** and **2**) were verified by the 100 ns MD simulation. The analysis of the MD simulation revealed that M24 and L133 are gatekeepers in the foot pocket of HDAC3. Additionally, the alkylhydrazides kept their bidentate chelation to the zinc ion during the 100 ns MD simulation. The compounds were rescored by means of BFE calculations. The binding free energies were correlated with the experimentally derived inhibitory activities. The established binding-free-energy-based QSAR model predicted all of the compounds in the test set with less than 1 log difference. Additionally, we tested the established model on a new dataset containing 26 molecules which were designed, synthesized, and tested taking knowledge of the developed BFE models [53]. The structure-based model was able to predict these novel compounds with less than 1 log unit error and showed its value for chemical optimization. For the different test sets, the structure-based model showed better accuracy than the ligand-based models. The combination of structure-based and ligand-based models resulted in predictive QSAR models in the current study. These provide useful tools for the further design and optimization of alkylhydrazide derivatives as HDAC inhibitors.

## Figures and Tables

**Table 1:** Distribution of inhibitors in the training and test sets according to their HDAC3 IC_50_ values.

HDAC3 Dataset	Number of Compounds	7 < pIC_50_ Highly Active	5.3 < pIC_50_ < 7 Moderately Active	pIC_50_ < 5.3 Inactive
Training	**39**	30	9	-
Test	**17**	11	6	-
Inactive	**7**	-	-	7
Total	**63**	41	15	7

**Table 2:** List of selected molecular descriptors for the PCA analysis.

Abbreviations	Molecular Descriptors
PEOE_VSA_HYD	The partial equalization of orbital electronegativity (PEOE). Total hydrophobic van der Waals surface area
GCUT_SLOGP_0	The GCUT descriptors using atomic contribution to logP
TPSA	Polar surface area
b_single	Number of single bonds (including implicit hydrogens). Aromatic bonds are not considered to be single bonds
lip_acc	The number of O and N atoms
lip_don	The number of OH and NH atoms
vsa_hyd	Approximation of the sum of VDW surface areas of hydrophobic atoms.

**Table 3:** Calculated scores of the best performing pharmacophore hypotheses.

HYPO ID	Survival Score	Inactive Score
ADDDHRR	6.923	1.688
DDDHRR	6.464	1.711
DDHR	5.405	2.069

**Table 4:** The best performing atom-based 3D_QSAR model.

HDAC3 Model	N	SD	R^2^	RMSE	Q^2^
1	39	0.27	0.95	0.39	0.88

Abbreviations: SD (standard deviation of the regression), R^2^ (correlation coefficient of the regression), RMSE (root mean square error of test set prediction), and Q^2^_LOO_ (leave one-out cross-validation for the prediction values).

**Table 5:** Prediction results of the test set compounds (atom-based QSAR model).

Compound Number	pIC_50_ HDAC3	Prediction by Atom-Based QSAR	Difference (Experimental—Predicted Activity) Atom-Based	References
**40**	9.29	8.69	0.60	[47]
**41**	9.24	8.87	0.37	[47]
**42**	9.21	9.14	0.07	[47]
**43**	7.90	8.43	0.53	[49]
**44**	7.78	7.13	0.64	[49]
**45**	7.46	7.17	0.29	[49]
**46**	7.31	7.65	0.34	[49]
**47**	7.17	6.92	0.24	[51]
**48**	7.16	7.54	0.38	[51]
**49**	7.11	7.96	0.85	[49]
**50**	7.07	7.81	0.74	[51]
**51**	6.73	7.88	1.16	[51]
**52**	6.51	7.32	0.81	[51]
**53**	5.96	7.85	1.89	[51]
**54**	5.87	6.19	0.33	[51]
**55**	5.81	8.08	2.26	[51]
**56**	5.72	6.74	1.01	[51]

**Table 6:** Best performing BFE models.

							LOO CV		3-Fold CV		10-Fold CV	
Model Number	N	Method	Frame	2D Descriptor	R^2^	RMSE	Q^2^	QMSE	Q^2^	QMSE	Q^2^	QMSE
MODEL1	39	GB1	Emin1	-	0.63	0.69	0.58	0.73	0.60	0.74	0.60	0.73
MODEL7	39	GB2	Emin1	-	0.66	0.65	0.63	0.69	0.64	0.69	0.64	0.69
MODEL13	39	GB5	Emin1	-	0.67	0.65	0.64	0.68	0.65	0.69	0.65	0.68
MODEL19	39	GB8	Emin1	-	0.81	0.49	0.78	0.52	0.78	0.54	0.77	0.53
MODEL19_1	39	GB8	Emin1	PEOE_VSA_HYD	0.87	0.40	0.84	0.44	0.85	0.45	0.83	0.45

Abbreviations: R^2^ (correlation coefficient), RMSE (root mean square error), Q^2^_LOO_ (leave one-out cross-validation), QMSE (crossed-root mean square error), and Emin1 (single frame after the first energy minimization step).

**Table 7:** Prediction results of the test set compounds using the BFE model (MODEL19_1).

Compound Number	pIC_50_ HDAC3	Prediction of BFE	Difference (Experimental—Predicted Activity)	References
**40**	9.29	8.60	0.70	[47]
**41**	9.24	9.75	0.51	[47]
**42**	9.21	9.37	0.17	[47]
**43**	7.90	8.53	0.63	[49]
**44**	7.78	8.10	0.33	[49]
**45**	7.46	7.42	0.03	[49]
**46**	7.31	8.06	0.75	[49]
**47**	7.17	7.85	0.68	[51]
**48**	7.16	8.12	0.96	[51]
**49**	7.11	6.30	0.81	[49]
**50**	7.07	7.32	0.25	[51]
**51**	6.73	7.56	0.83	[51]
**52**	6.51	6.89	0.38	[51]
**53**	5.96	6.19	0.22	[51]
**54**	5.87	6.41	0.55	[51]
**55**	5.81	6.27	0.46	[51]
**56**	5.72	6.42	0.69	[51]

**Table 8:** Experimental and predicted activities of the BFE-based and atom-based models.

Compound Number	pIC_50_ HDAC3	Prediction by Atom-Based QSAR	Difference (Experimental—Predicted Activity) Atom-Based	Prediction by BFE Model	Difference (Experimental—Predicted Activity) BFE
**64**	7.04	7.03	0.01	7.16	−0.12
**65**	6.46	6.59	−0.13	6.89	−0.43
**66**	5.82	6.57	−0.75	6.36	−0.54
**67**	<5.00	6.43	<−1.43	5.41	-
**68**	5.80	7.25	−1.46	6.69	−0.89
**69**	<5.00	7.05	<−2.05	5.07	-
**70**	<5.00	6.98	<−1.98	3.92	-
**71**	9%@1 µM	6.82	-	0.24	-
**72**	7.37	7.85	−0.49	7.60	−0.23
**73**	6.70	7.29	−0.59	6.68	0.02
**74**	41%@1 µM	7.39	-	7.29	-
**75**	7.09	7.82	−0.73	8.06	−0.97
**76**	7.22	7.12	0.10	6.86	0.36
**77**	7.43	7.96	−0.53	7.06	0.38
**78**	7.24	7.03	0.20	7.98	−0.75
**79**	6.92	6.80	0.12	6.32	0.60
**80**	6.96	8.10	−1.14	7.42	−0.46
**81**	<5.00	6.54	<−1.54	1.65	-
**82**	<5.00	6.86	<1.86	1.61	-
**83**	5.52	7.25	−1.73	3.26	2.26
**84**	<5.00	7.61	<−1.67	2.69	-
**85**	7.52	7.63	−0.10	7.64	−0.11
**86**	7.00	7.18	−0.18	7.29	−0.29
**87**	6.52	6.52	0.00	6.86	−0.33
**88**	6.00	7.40	−1.40	6.13	−0.13
**89**	5.85	7.13	−1.28	6.11	−0.26

## Data Availability

Not applicable.

## Supplementary Materials

The following supporting information can be downloaded at: https://www.mdpi.com/article/10.3390/ph16070968/s1. Figure S1: Visualization of the atom-based 3D-QSAR model in the training set; Figure S2: Docking poses of the training set, the external test set, and the inactive set; Figure S3: 100 ns MD results for the HDAC3-1 complex; Figure S4: 100 ns MD results for the HDAC3-2 complex; Figure S5: MD analysis results of HDAC3-1 complex. Figure S6: MD analysis results of HDAC3-2 complex. Table S1: Two-dimensional chemical structures and biological data of compounds in the training set, external test set, and inactive set; Table S2: Prediction values of the best atom-based QSAR model generated for the training set, test set, and inactive set in HDAC3; Table S3: Prediction values of the best BFE model generated for the training set, test set, and inactive set in HDAC3; Table S4: Two-dimensional chemical structures and biological data of the test set; Table S5: Prediction values of the best atom-based QSAR model generated for the test set in HDAC3; Table S6: Prediction values of the best BFE model generated for the test set in HDAC3.

## References

[1] Weinhold B. (2006). Epigenetics: The science of change. Environ. Health Perspect..

[2] Kouzarides T. (2007). Chromatin modifications and their function. Cell.

[3] Fraczek J., van Grunsven L.A., Vinken M., Snykers S., Deleu S., Vanderkerken K., Vanhaecke T., Rogiers V. (2009). Histone deacetylase inhibition and the regulation of cell growth with particular reference to liver pathobiology. J. Cell. Mol. Med..

[4] Chen P.J., Huang C., Meng X.M., Li J. (2015). Epigenetic modifications by histone deacetylases: Biological implications and therapeutic potential in liver fibrosis. Biochimie.

[5] Haberland M., Montgomery R.L., Olson E.N. (2009). The many roles of histone deacetylases in development and physiology: Implications for disease and therapy. Nat. Rev. Genet..

[6] Seto E., Yoshida M. (2014). Erasers of Histone Acetylation: The Histone Deacetylase Enzymes. Cold Spring Harb. Perspect. Biol..

[7] Melesina J., Simoben C.V., Praetorius L., Bulbul E.F., Robaa D., Sippl W. (2021). Strategies To Design Selective Histone Deacetylase Inhibitors. Chemmedchem.

[8] Bolden J.E., Peart M.J., Johnstone R.W. (2006). Anticancer activities of histone deacetylase inhibitors. Nat. Rev. Drug Discov..

[9] Gregoretti I.V., Lee Y.M., Goodson H.V. (2004). Molecular evolution of the histone deacetylase family: Functional implications of phylogenetic analysis. J. Mol. Biol..

[10] Hildmann C., Riester D., Schwienhorst A. (2007). Histone deacetylases--an important class of cellular regulators with a variety of functions. Appl. Microbiol. Biotechnol..

[11] Barneda-Zahonero B., Parra M. (2012). Histone deacetylases and cancer. Mol. Oncol..

[12] Denslow S.A., Wade P.A. (2007). The human Mi-2/NuRD complex and gene regulation. Oncogene.

[13] Grozinger C.M., Schreiber S.L. (2002). Deacetylase enzymes: Biological functions and the use of small-molecule inhibitors. Chem. Biol..

[14] Laherty C.D., Yang W.M., Sun J.M., Davie J.R., Seto E., Eisenman R.N. (1997). Histone deacetylases associated with the mSin3 corepressor mediate Mad transcriptional repression. Cell.

[15] Turnbull R.E., Fairall L., Saleh A., Kelsall E., Morris K.L., Ragan T.J., Savva C.G., Chandru A., Millard C.J., Makarova O.V. (2020). The MiDAC histone deacetylase complex is essential for embryonic development and has a unique multivalent structure. Nat. Commun..

[16] Xue Y.T., Wong J.M., Moreno G.T., Young M.K., Cote J., Wang W.D. (1998). NURD, a novel complex with both ATP-dependent chromatin-remodeling and histone deacetylase activities. Mol. Cell.

[17] Li J.W., Wang J., Wang J.X., Nawaz Z., Liu J.M., Qin J., Wong J.M. (2000). Both corepressor proteins SMRT and N-CoR exist in large protein complexes containing HDAC3. EMBO J..

[18] Oberoi J., Fairall L., Watson P.J., Yang J.C., Czimmerer Z., Kampmann T., Goult B.T., Greenwood J.A., Gooch J.T., Kallenberger B.C. (2011). Structural basis for the assembly of the SMRT/NCoR core transcriptional repression machinery. Nat. Struct. Mol. Biol..

[19] Hu E., Chen Z.X., Fredrickson T., Zhu Y., Kirkpatrick R., Zhang G.F., Johanson K., Sung C.M., Liu R.G., Winkler J. (2000). Cloning and characterization of a novel human. Class I histone deacetylase that functions as a transcription repressor. J. Biol. Chem..

[20] Park S.Y., Kim J.S. (2020). A short guide to histone deacetylases including recent progress on class II enzymes. Exp. Mol. Med..

[21] Vahid F., Zand H., Nosrat-Mirshekarlou E., Najafi R., Hekmatdoost A. (2015). The role dietary of bioactive compounds on the regulation of histone acetylases and deacetylases: A review. Gene.

[22] Chien W.W., Lee D.H., Zheng Y., Wuensche P., Alvarez R., Wen D.L., Aribi A.M., Thean S.M., Doan N.B., Said J.W. (2014). Growth Inhibition of Pancreatic Cancer Cells by Histone Deacetylase Inhibitor Belinostat Through Suppression of Multiple Pathways Including HIF, NFkB, and mTOR Signaling In Vitro and In Vivo. Mol. Carcinogen..

[23] Furumai R., Matsuyama A., Kobashi N., Lee K.H., Nishiyama N., Nakajima I., Tanaka A., Komatsu Y., Nishino N., Yoshida M. (2002). FK228 (depsipeptide) as a natural prodrug that inhibits class I histone deacetylases. Cancer Res..

[24] Mann B.S., Johnson J.R., He K., Sridhara R., Abraham S., Booth B.P., Verbois L., Morse D.E., Jee J.M., Pope S. (2007). Vorinostat for treatment of cutaneous manifestations of advanced primary cutaneous T-cell lymphoma. Clin. Cancer Res..

[25] Sivaraj D., Green M.M., Gasparetto C. (2017). Panobinostat for the management of multiple myeloma. Future Oncol..

[26] Fraga M.F., Ballestar E., Villar-Garea A., Boix-Chornet M., Espada J., Schotta G., Bonaldi T., Haydon C., Ropero S., Petrie K. (2005). Loss of acetylation at Lys16 and trimethylation at Lys20 of histone H4 is a common hallmark of human cancer. Nat. Genet..

[27] Gryder B.E. (2012). Targeted cancer therapy: Giving histone deacetylase inhibitors all they need to succeed. Future Med. Chem..

[28] Hailu G.S., Robaa D., Forgione M., Sippl W., Rotili D., Mai A. (2017). Lysine Deacetylase Inhibitors in Parasites: Past, Present, and Future Perspectives. J. Med. Chem..

[29] Pant K., Peixoto E., Richard S., Gradilone S.A. (2020). Role of Histone Deacetylases in Carcinogenesis: Potential Role in Cholangiocarcinoma. Cells.

[30] Jung M., Brosch G., Kolle D., Scherf H., Gerhauser C., Loidl P. (1999). Amide analogues of trichostatin A as inhibitors of histone deacetylase and inducers of terminal cell differentiation. J. Med. Chem..

[31] Bulbul E.F., Melesina J., Ibrahim H.S., Abdelsalam M., Vecchio A., Robaa D., Zessin M., Schutkowski M., Sippl W. (2022). Docking, Binding Free Energy Calculations and In Vitro Characterization of Pyrazine Linked 2-Aminobenzamides as Novel Class I Histone Deacetylase (HDAC) Inhibitors. Molecules.

[32] Burli R.W., Luckhurst C.A., Aziz O., Matthews K.L., Yates D., Lyons K.A., Beconi M., McAllister G., Breccia P., Stott A.J. (2013). Design, Synthesis, and Biological Evaluation of Potent and Selective Class IIa Histone Deacetylase (HDAC) Inhibitors as a Potential Therapy for Huntington’s Disease. J. Med. Chem..

[33] Heimburg T., Chakrabarti A., Lancelot J., Marek M., Melesina J., Hauser A.T., Shaik T.B., Duclaud S., Robaa D., Erdmann F. (2016). Structure-Based Design and Synthesis of Novel Inhibitors Targeting HDAC8 from Schistosoma mansoni for the Treatment of Schistosomiasis. J. Med. Chem..

[34] Ibrahim H.S., Abdelsalam M., Zeyn Y., Zessin M., Mustafa A.M., Fischer M.A., Zeyen P., Sun P., Bulbul E.F., Vecchio A. (2022). Synthesis, Molecular Docking and Biological Characterization of Pyrazine Linked 2-Aminobenzamides as New Class I Selective Histone Deacetylase (HDAC) Inhibitors with Anti-Leukemic Activity. Int. J. Mol. Sci..

[35] Lauffer B.E., Mintzer R., Fong R., Mukund S., Tam C., Zilberleyb I., Flicke B., Ritscher A., Fedorowicz G., Vallero R. (2013). Histone deacetylase (HDAC) inhibitor kinetic rate constants correlate with cellular histone acetylation but not transcription and cell viability. J. Biol. Chem..

[36] Luckhurst C.A., Breccia P., Stott A.J., Aziz O., Birch H.L., Burli R.W., Hughes S.J., Jarvis R.E., Lamers M., Leonard P.M. (2016). Potent, Selective, and CNS-Penetrant Tetrasubstituted Cyclopropane Class Ila Histone Deacetylase (HDAC) Inhibitors. ACS Med. Chem. Lett..

[37] Marek M., Ramos-Morales E., Picchi-Constante G.F.A., Bayer T., Norstrom C., Herp D., Sales P.A., Guerra-Slompo E.P., Hausmann K., Chakrabarti A. (2021). Species-selective targeting of pathogens revealed by the atypical structure and active site of Trypanosoma cruzi histone deacetylase DAC2. Cell Rep..

[38] Marek M., Shaik T.B., Heimburg T., Chakrabarti A., Lancelot J., Ramos-Morales E., Da Veiga C., Kalinin D., Melesina J., Robaa D. (2018). Characterization of Histone Deacetylase 8 (HDAC8) Selective Inhibition Reveals Specific Active Site Structural and Functional Determinants. J. Med. Chem..

[39] Simoben C.V., Robaa D., Chakrabarti A., Schmidtkunz K., Marek M., Lancelot J., Kannan S., Melesina J., Shaik T.B., Pierce R.J. (2018). A Novel Class of Schistosoma mansoni Histone Deacetylase 8 (HDAC8) Inhibitors Identified by Structure-Based Virtual Screening and In Vitro Testing. Molecules.

[40] Wang D.F., Wiest O., Helquist P., Lan-Hargest H.Y., Wiech N.L. (2004). On the function of the 14 angstrom long internal cavity of histone deacetylase-like protein: Implications for the design of histone deacetylase inhibitors. J. Med. Chem..

[41] Bressi J.C., Jennings A.J., Skene R., Wu Y.Q., Melkus R., De Jong R., O’Connell S., Grimshaw C.E., Navre M., Gangloff A.R. (2010). Exploration of the HDAC2 foot pocket: Synthesis and SAR of substituted N-(2-aminophenyl)benzamides. Bioorg. Med. Chem. Lett..

[42] Liu J., Kelly J., Yu W.S., Clausen D., Yu Y.N., Kim H., Duffy J.L., Chung C.C., Myers R.W., Carroll S. (2020). Selective Class I HDAC Inhibitors Based on Aryl Ketone Zinc Binding Induce HIV-1 Protein for Clearance. ACS Med. Chem. Lett..

[43] Wagner F.F., Weiwer M., Steinbacher S., Schomburg A., Reinemer P., Gale J.P., Campbell A.J., Fisher S.L., Zhao W.N., Reis S.A. (2016). Kinetic and structural insights into the binding of histone deacetylase 1 and 2 (HDAC1, 2) inhibitors. Bioorgan. Med. Chem..

[44] Yu W.S., Liu J., Yu Y.N., Zhang V., Clausen D., Kelly J., Wolkenberg S., Beshore D., Duffy J.L., Chung C.C. (2020). Discovery of ethyl ketone-based HDACs 1, 2, and 3 selective inhibitors for HIV latency reactivation. Bioorg. Med. Chem. Lett..

[45] Liu J., Yu Y.N., Kelly J., Sha D.Y., Alhassan A.B., Yu W.S., Maletic M.M., Duffy J.L., Klein D.J., Holloway M.K. (2020). Discovery of Highly Selective and Potent HDAC3 Inhibitors Based on a 2-Substituted Benzamide Zinc Binding Group. ACS Med. Chem. Lett..

[46] Wang Y.F., Stowe R.L., Pinello C.E., Tian G.M., Madoux F., Li D.W., Zhao L.S.Y., Li J.L., Wang Y.R., Wang Y. (2015). Identification of Histone Deacetylase Inhibitors with Benzoylhydrazide Scaffold that Selectively Inhibit Class I Histone Deacetylases. Chem. Biol..

[47] Jiang Y.Q., Xu J., Yue K.R., Huang C., Qin M.T., Chi D.Y., Yu Q.X., Zhu Y., Hou X.H., Xu T.Q. (2022). Potent Hydrazide-Based HDAC Inhibitors with a Superior Pharmacokinetic Profile for Efficient Treatment of Acute Myeloid Leukemia In Vivo. J. Med. Chem..

[48] Kozlov M.V., Konduktorov K.A., Shcherbakova A.S., Kochetkov S.N. (2019). Synthesis of *N*′-propylhydrazide analogs of hydroxamic inhibitors of histone deacetylases (HDACs) and evaluation of their impact on activities of HDACs and replication of hepatitis C virus (HCV). Bioorg. Med. Chem. Lett..

[49] Li X.Y., Jiang Y.Q., Peterson Y.K., Xu T.Q., Himes R.A., Luo X., Yin G.L., Inks E.S., Dolloff N., Halene S. (2020). Design of Hydrazide-Bearing HDACIs Based on Panobinostat and Their p53 and FLT3-ITD Dependency in Antileukemia Activity. J. Med. Chem..

[50] Li X.Y., Peterson Y.K., Inks E.S., Himes R.A., Li J.Y., Zhang Y.J., Kong X.J., Chou C.J. (2018). Class I HDAC Inhibitors Display Different Antitumor Mechanism in Leukemia and Prostatic Cancer Cells Depending on Their p53 Status. J. Med. Chem..

[51] McClure J.J., Zhang C., Inks E.S., Peterson Y.K., Li J.Y., Chou C.J. (2016). Development of Allosteric Hydrazide-Containing Class I Histone Deacetylase Inhibitors for Use in Acute Myeloid Leukemia. J. Med. Chem..

[52] Xiao Y.F., Wang J., Zhao L.S.Y., Chen X.Y., Zheng G.R., Zhang X., Liao D.Q. (2020). Discovery of histone deacetylase 3 (HDAC3)-specific PROTACs. Chem. Commun..

[53] Sun P., Wang J., Khan K.S., Yang W., Ng B.W.-L., Ilment N., Zessin M., Bülbül E.F., Robaa D., Erdmann F. (2022). Development of alkylated hydrazides as highly potent and selective class I HDAC inhibitors with T cell modulatory properties. J. Med. Chem..

[54] Adhikari N., Jha T., Ghosh B. (2021). Dissecting Histone Deacetylase 3 in Multiple Disease Conditions: Selective Inhibition as a Promising Therapeutic Strategy. J. Med. Chem..

[55] You S.H., Lim H.W., Sun Z., Broache M., Won K.J., Lazar M.A. (2013). Nuclear receptor co-repressors are required for the histone-deacetylase activity of HDAC3 in vivo. Nat. Struct. Mol. Biol..

[56] Sarkar R., Banerjee S., Amin S.A., Adhikari N., Jha T. (2020). Histone deacetylase 3 (HDAC3) inhibitors as anticancer agents: A review. Eur. J. Med. Chem..

[57] Janczura K.J., Volmar C.H., Sartor G.C., Rao S.J., Ricciardi N.R., Lambert G., Brothers S.P., Wahlestedt C. (2018). Inhibition of HDAC3 reverses Alzheimer’s disease-related pathologies in vitro and in the 3xTg-AD mouse model. Proc. Natl. Acad. Sci. USA.

[58] Jiang L.P., Yu X.H., Chen J.Z., Hu M., Zhang Y.K., Lin H.L., Tang W.Y., He P.P., Ouyang X.P. (2022). Histone Deacetylase 3: A Potential Therapeutic Target for Atherosclerosis. Aging Dis..

[59] Zhang L., Cao W. (2022). Histone deacetylase 3 (HDAC3) as an important epigenetic regulator of kidney diseases. J. Mol. Med..

[60] Carey R.N., Wold S., Westgard J.O. (1975). Principal component analysis: An alternative to “referee” methods in method comparison studies. Anal. Chem..

[61] Eichner L.J., Curtis S.D., Brun S.N., McGuire C.K., Gushterova I., Baumgart J.T., Trefts E., Ross D.S., Rymoff T.J., Shaw R.J. (2023). HDAC3 is critical in tumor development and therapeutic resistance in Kras-mutant non-small cell lung cancer. Sci. Adv..

[62] Jia H., Wang Y., Morris C.D., Jacques V., Gottesfeld J.M., Rusche J.R., Thomas E.A. (2016). The Effects of Pharmacological Inhibition of Histone Deacetylase 3 (HDAC3) in Huntington’s Disease Mice. PLoS ONE.

[63] Montgomery R.L., Potthoff M.J., Haberland M., Qi X., Matsuzaki S., Humphries K.M., Richardson J.A., Bassel-Duby R., Olson E.N. (2008). Maintenance of cardiac energy metabolism by histone deacetylase 3 in mice. J. Clin. Investig..

[64] Chemical Computing Group (CCG) (2019). Molecular Operating Environment (MOE), 2019.01.

[65] Jolliffe I.T., Cadima J. (2016). Principal component analysis: A review and recent developments. Philos. Trans. R. Soc. A.

[66] Schrödinger LLC (2019). Release 2019-1: Phase.

[67] Dixon S.L., Smondyrev A.M., Knoll E.H., Rao S.N., Shaw D.E., Friesner R.A. (2006). PHASE: A new engine for pharmacophore perception, 3D QSAR model development, and 3D database screening: 1. Methodology and preliminary results. J. Comput.-Aid. Mol. Des..

[68] Dixon S.L., Smondyrev A.M., Rao S.N. (2006). PHASE: A novel approach to pharmacophore modeling and 3D database searching. Chem. Biol. Drug Des..

[69] Watson P.J., Fairall L., Santos G.M., Schwabe J.W.R. (2012). Structure of HDAC3 bound to co-repressor and inositol tetraphosphate. Nature.

[70] Case D.A., Cheatham T.E., Darden T., Gohlke H., Luo R., Merz K.M., Onufriev A., Simmerling C., Wang B., Woods R. (2005). The Amber biomolecular simulation programs. J. Comput. Chem..

[71] Tropsha A. (2010). Best Practices for QSAR Model Development, Validation, and Exploitation. Mol. Inform..

[72] Roe D.R., Okur A., Wickstrom L., Hornak V., Simmerling C. (2007). Secondary structure bias in generalized Born solvent models: Comparison of conformational ensembles and free energy of solvent polarization from explicit and implicit solvation. J. Phys. Chem. B.

[73] Schrödinger LLC (2019). Release 2019-1: Maestro, Protein Preparation Wizard, Prime, Epik, Ligprep, Confgen, Glide.

[74] Harder E., Damm W., Maple J., Wu C.J., Reboul M., Xiang J.Y., Wang L.L., Lupyan D., Dahlgren M.K., Knight J.L. (2016). OPLS3: A Force Field Providing Broad Coverage of Drug-like Small Molecules and Proteins. J. Chem. Theory Comput..

[75] Berman H.M., Westbrook J., Feng Z., Gilliland G., Bhat T.N., Weissig H., Shindyalov I.N., Bourne P.E. (2000). The Protein Data Bank. Nucleic Acids Res..

[76] Jakalian A., Jack D.B., Bayly C.I. (2002). Fast, efficient generation of high-quality atomic charges. AM1-BCC model: II. Parameterization and validation. J. Comput. Chem..

[77] Jakalian A., Bush B.L., Jack D.B., Bayly C.I. (2000). Fast, efficient generation of high-quality atomic Charges. AM1-BCC model: I. Method. J. Comput. Chem..

[78] Wang J.M., Wolf R.M., Caldwell J.W., Kollman P.A., Case D.A. (2004). Development and testing of a general amber force field. J. Comput. Chem..

[79] Li P.F., Song L.F., Merz K.M. (2015). Parameterization of Highly Charged Metal Ions Using the 12-6-4 LJ-Type Nonbonded Model in Explicit Water. J. Phys. Chem. B.

[80] Lee M.C., Duan Y. (2004). Distinguish protein decoys by using a scoring function based on a new AMBER force field, short molecular dynamics simulations, and the generalized born solvent model. Proteins-Struct. Funct. Bioinform..

[81] Duan Y., Wu C., Chowdhury S., Lee M.C., Xiong G.M., Zhang W., Yang R., Cieplak P., Luo R., Lee T. (2003). A point-charge force field for molecular mechanics simulations of proteins based on condensed-phase quantum mechanical calculations. J. Comput. Chem..

[82] Ryckaert J.-P., Ciccotti G., Berendsen H.J.C. (1977). Numerical integration of the cartesan equations of motion of a system with constraints: Molecular dynamics of n-alkanes. J. Comput. Phys..

[83] Pastor R.W., Brooks B.R., Szabo A. (1988). An analysis of the accuracy of Langevin and molecular dynamics algorithms. Mol. Phys..

[84] Miller B.R., McGee T.D., Swails J.M., Homeyer N., Gohlke H., Roitberg A.E. (2012). MMPBSA.py: An Efficient Program for End-State Free Energy Calculations. J. Chem. Theory Comput..

[85] Onufriev A., Bashford D., Case D.A. (2004). Exploring protein native states and large-scale conformational changes with a modified generalized born model. Proteins-Struct. Funct. Bioinform..

[86] Hawkins G.D., Cramer C.J., Truhlar D.G. (1996). Parametrized models of aqueous free energies of solvation based on pairwise descreening of solute atomic charges from a dielectric medium. J. Phys. Chem..

[87] Feig M., Onufriev A., Lee M.S., Im W., Case D.A., Brooks C.L. (2004). Performance comparison of generalized born and Poisson methods in the calculation of electrostatic solvation energies for protein structures. J. Comput. Chem..

[88] Karaman B., Sippl W. (2015). Docking and binding free energy calculations of sirtuin inhibitors. Eur. J. Med. Chem..

[89] Genheden S., Ryde U. (2015). The MM/PBSA and MM/GBSA methods to estimate ligand-binding affinities. Expert Opin. Drug Dis..

[90] Cournia Z., Allen B., Sherman W. (2017). Relative Binding Free Energy Calculations in Drug Discovery: Recent Advances and Practical Considerations. J. Chem. Inf. Model..

