# Supplementary material for: Application of Ligand- and Structure-Based Prediction Models for the Design of Alkylhydrazide-Based HDAC3 Inhibitors as Novel Anti-Cancer Compounds
Source: Pharmaceuticals (Basel). 2023 Jul 6;16(7):968. doi: 10.3390/ph16070968 (PMC10386743; doi:10.3390/ph16070968)
Supplement: Supplementary file 1 [file pharmaceuticals-16-00968-s001.zip › pharmaceuticals-2425558-supplementary.pdf]

Application of Ligand- and Structure-Based Prediction Models for the Design of Alkylhydrazide-Based HDAC3 Inhibitors as Novel Anti-Cancer Compounds

Emre F. Bülbül [1], Dina Robaa [1], Ping Sun [1], Fereshteh Mahmoudi [1], Jelena Melesina [1], Matthes Zessin [2], Mike Schutkowski [2] and Wolfgang Sippl [1,*]

Table of contents

	Topic	Page
1	**Figure S1**. Atom-based 3D-QSAR models for the training set	**S2**
2	**Figure S2**. Docking poses of training, external test and inactive set	**S3**
3	**Figure S3.** 100 ns MD results for the HDAC3- compound **1** complex	**S4**
4	**Figure S4.** Analysis of hydrogen bond distances for the HDAC3- compound **1** complex from 100 ns MD simulation	**S5**
5	**Figure S5.** 100 ns MD results for the HDAC3- compound **2** complex	**S6**
6	**Figure S6.** Analysis of hydrogen bond distances for the HDAC3- compound **2** complex from 100 ns MD simulation	**S7**
7	**Table S1**. 2D chemical structures and biological data of compounds in training set, external test set and inactive set	**S8**
8	**Table S2**. Prediction values of the best atom-based QSAR model generated for training set, test set and inactive set for HDAC3	**S16**
9	**Table S3**. Prediction values of the best BFE model generated for training set, test set and inactive set for HDAC3	**S19**
10	**Table S4.** 2D chemical structures and biological data of different test set	**S22**
11	**Table S5**. Prediction values of the best atom-based QSAR model generated for test set for HDAC3	**S27**
12	**Table S6**. Prediction values of the best BFE model generated for test set for HDAC3	**S29**

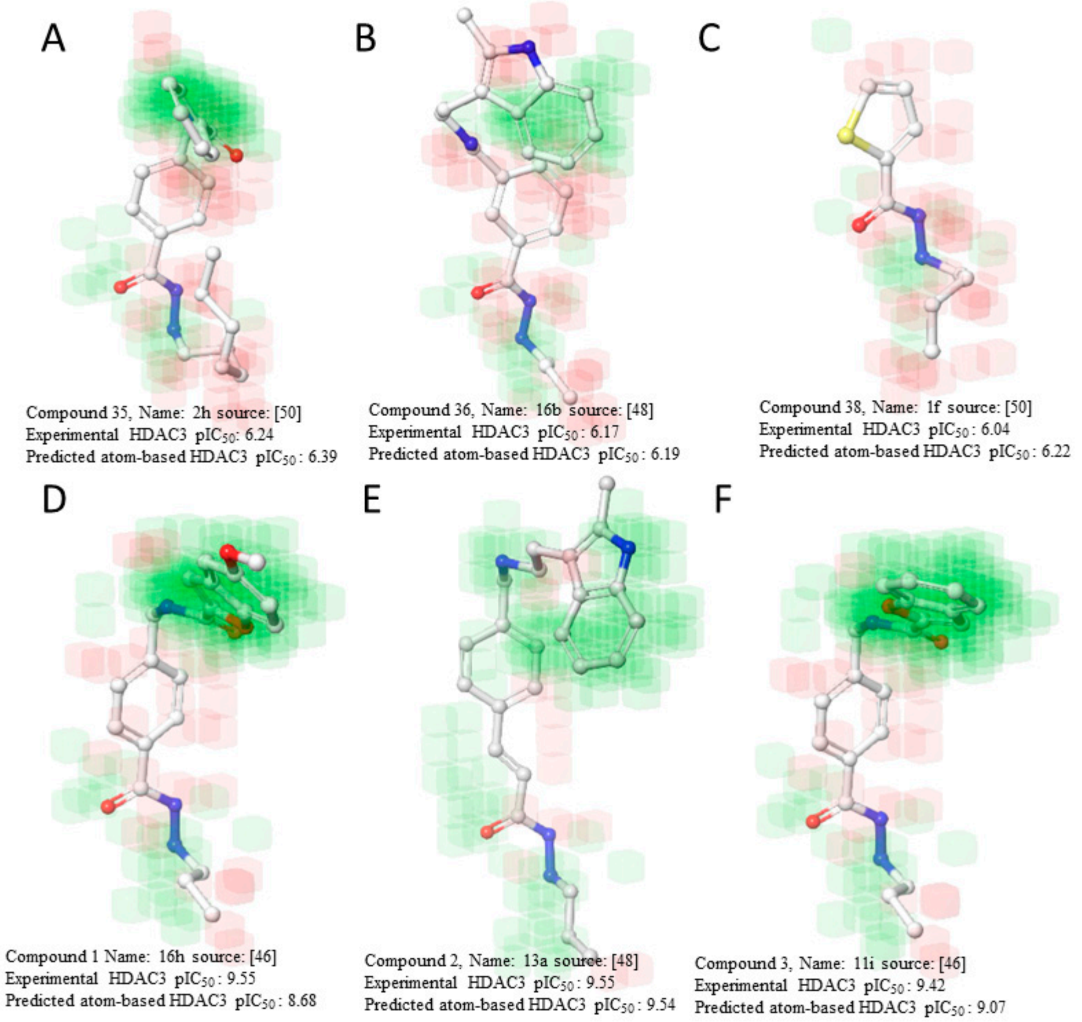

Figure S1. Visualization of Atom-based 3D-QSAR model in training set. A) Compound 35 [50] B) Compound 36 [48] , C) Compound 38 [50], D) Compound 1 [46] E) Compound 2 [48] F) Compound 3 [46]. Favorable regions shown as green color, unfavorable regions as red color.

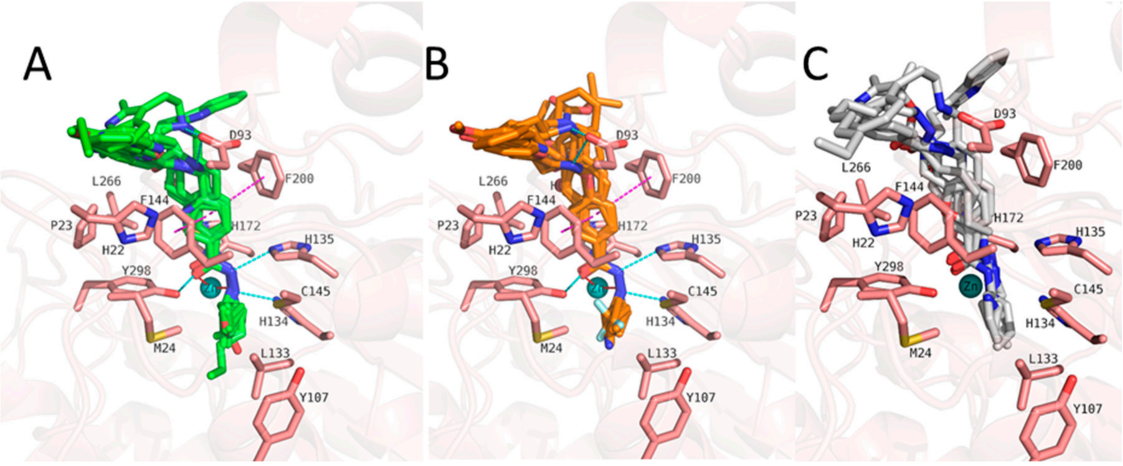

Figure S2. Docking poses of **training set** (39 compounds, A, green colored sticks), **external test set** (17 compounds, B, orange colored sticks), **inactive set** (7 compounds, C, gray colored sticks) in HDAC3 (PDB ID: 4A69). Hydrogen bonds (cyan dashed lines), hydrophobic interactions (magenta dashed lines) and metal coordination (red dashed lines) between inhibitors and protein are shown. Relevant residues are shown in stick representation with salmon carbon atoms in HDAC3. The zinc ion is shown as cyan colored sphere.

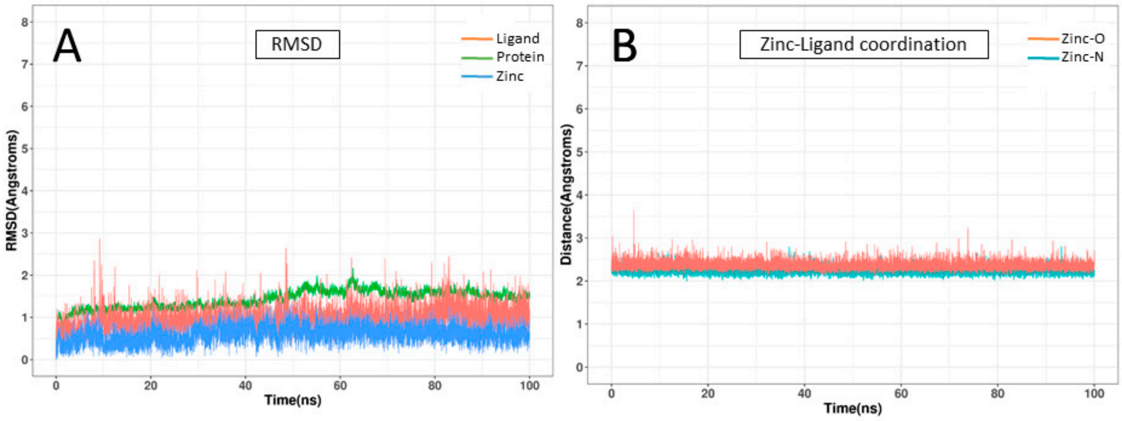

Figure S3. 100 ns MD results for the HDAC3- compound **1** complex (PDB ID: 4A69). A) RMSD plot for the HDAC3- compound **1** complex. The ligand is represented as red line, the backbone atoms of the protein as green line, and the zinc ion as blue line. B) The distance between zinc and free amino group of ligand (cyan lines) and the distance between zinc and carbonyl oxygen of ligand (red lines)

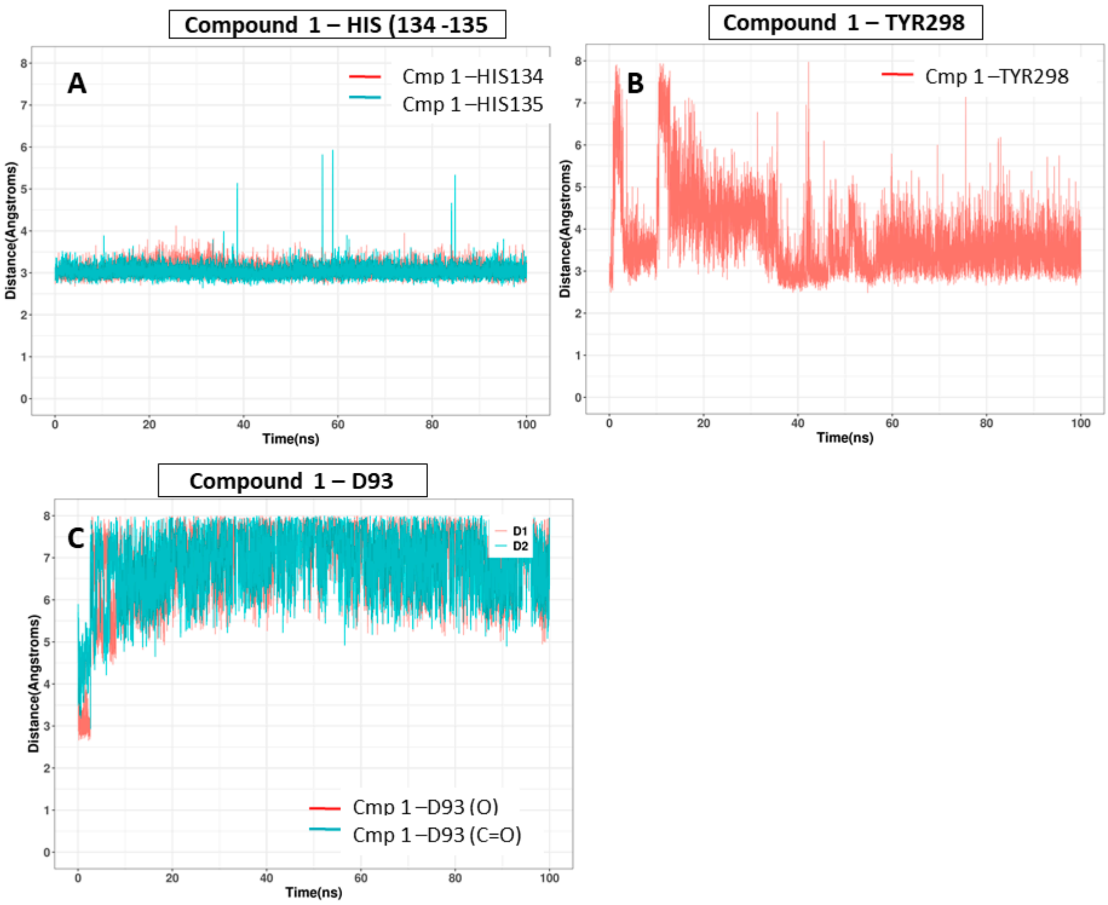

Figure S4. Hydrogen bond distance analysis of 100ns MD results for the HDAC3-**1** complex. A) RMSD plot for the HDAC3- compound **1** complex. A) Distance analysis of compound **1** – HIS134 (red lines) and compound **1** – HIS135 (cyan lines) B) Distance analysis of ligand – TYR298 (red lines) and compound **1** – HIS135 (cyan lines). C) Distance analysis of compound **1** – D93 (O – red lines, C=O cyan lines).

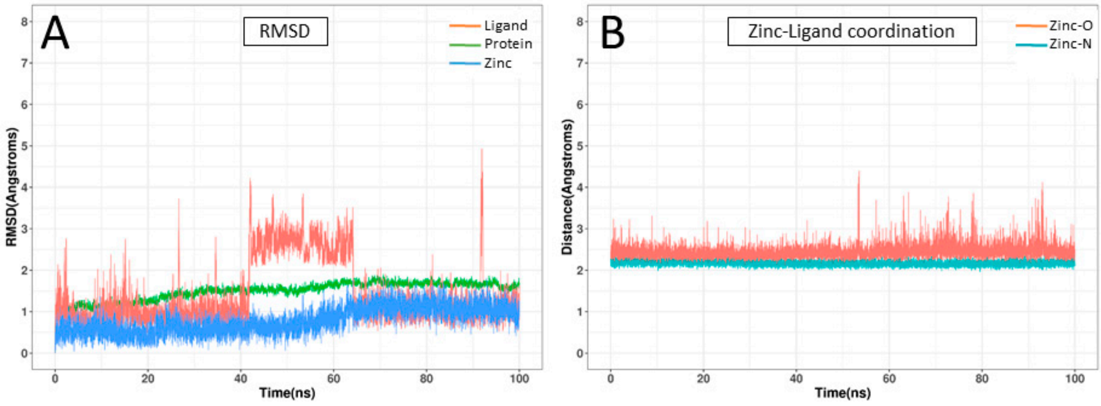

Figure S5. 100 ns MD results for the HDAC3- compound **2** complex (PDB ID: 4A69). A) RMSD plot for the HDAC3- compound **2** complex. A) The ligand is represented as red line, the backbone atoms of the protein as green line, and the zinc ion as blue line. B) The distance between zinc and free amino group of ligand (cyan lines) and the distance between zinc and carbonyl oxygen of ligand (red lines)

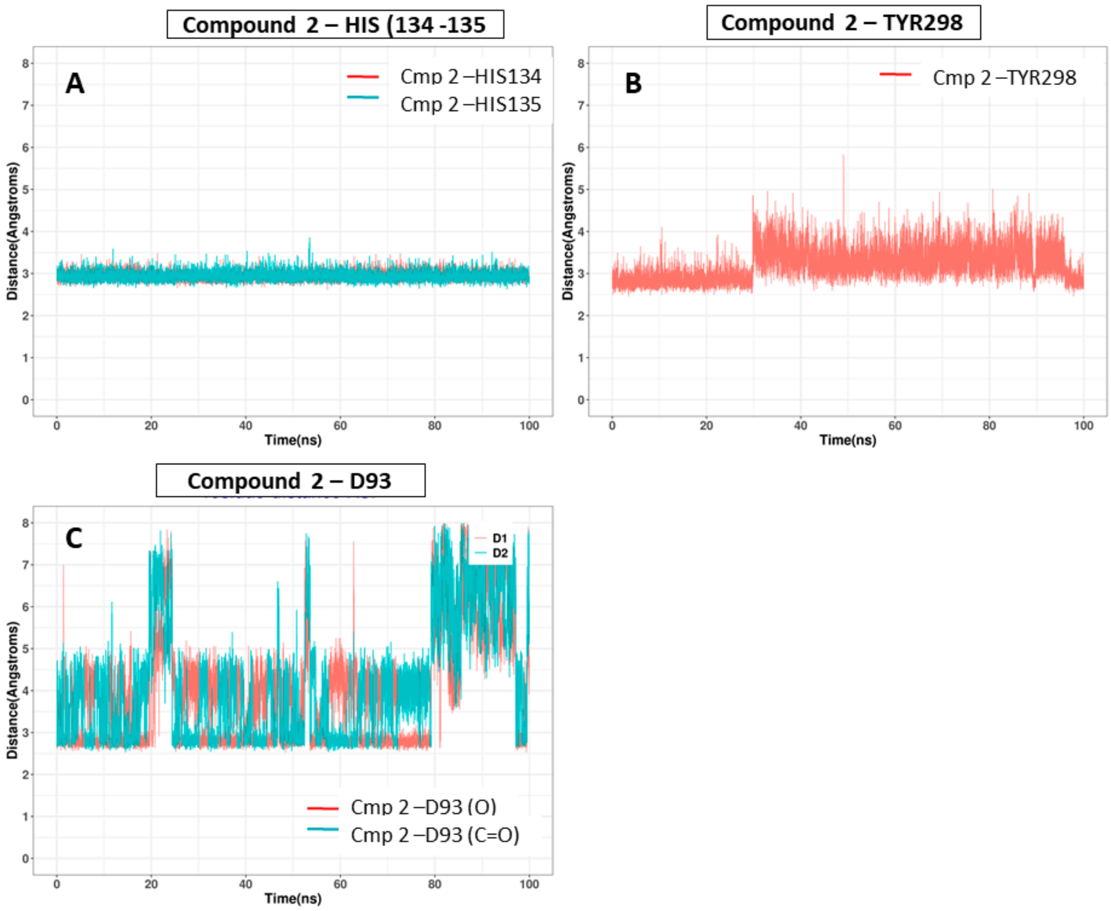

Figure S6. Hydrogen bond distance analysis of 100 ns MD results for the HDAC3-**2** complex (PDB ID: 4A69). A) RMSD plot for the HDAC3-**2** complex. A) Distance analysis of compound **2** – HIS134 (red lines) and compound **2** – HIS135 (cyan lines) B) Distance analysis of compound **2** – TYR298 (red lines) and compound **2** – HIS135 (cyan lines). C) Distance analysis of compound **2** – D93 (O – red lines, C=O cyan lines).

Table S1. 2D chemical structures and biological data of compounds in training set, external test set and inactive set.

Compound number	Molecular structure – ID in the original article	Class	HDAC3 experimental pIC_{50}	Ref.
1	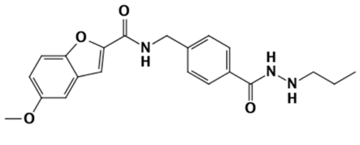 16h	training	9.55	[46]
2	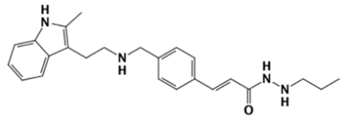 13a	training	9.55	[48]
3	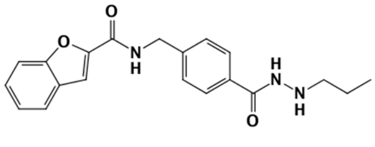 11i	training	9.42	[46]
4	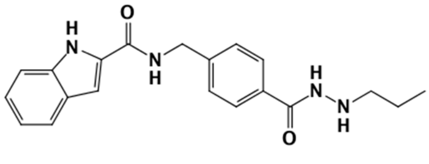 11h	training	9.36	[46]
5	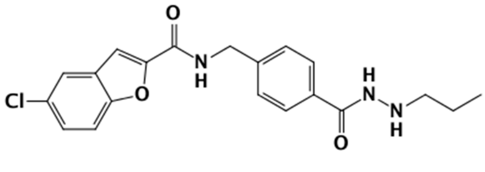 16f	training	9.21	[46]
6	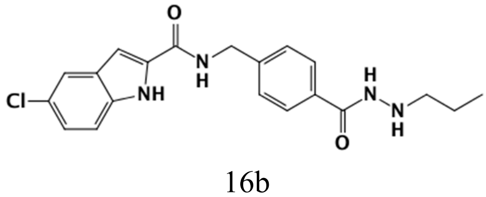 16b	training	9.18	[46]
7	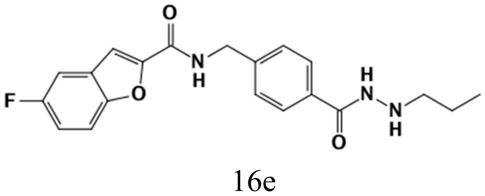 16e	training	9.08	[46]

8	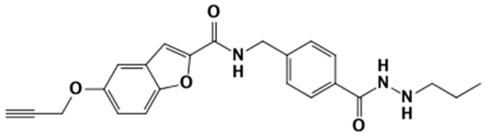<br>11n	training	9.07	[46]
9	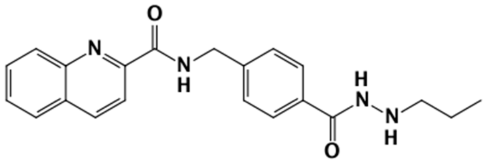<br>11d	training	9.05	[46]
10	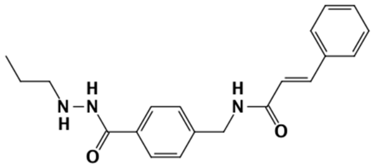<br>3b	training	9.02	[50]
11	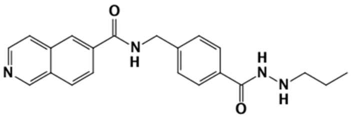<br>11e	training	8.99	[46]
12	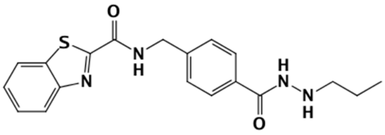<br>11l	training	8.88	[46]
13	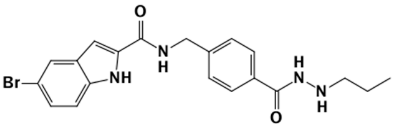<br>16c	training	8.88	[46]
14	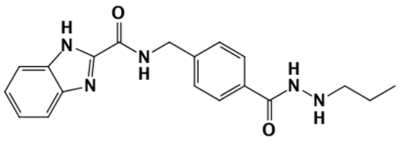<br>11k	training	8.84	[46]
15	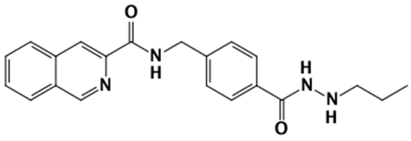<br>11c	training	8.83	[46]

16	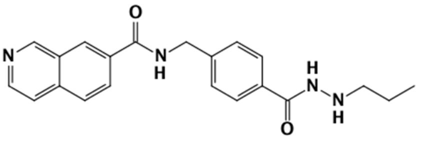 11f	training	8.81	[46]
17	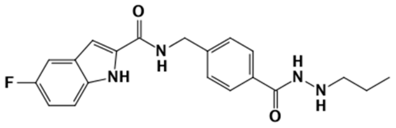 16a	training	8.79	[46]
18	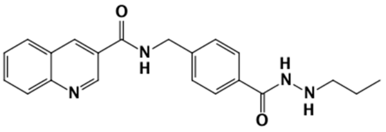 11b	training	8.75	[46]
19	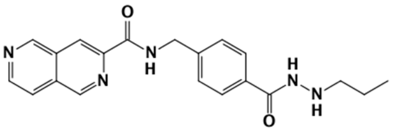 11g	training	8.69	[46]
20	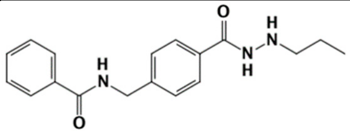 2d	training	8.46	[50]
21	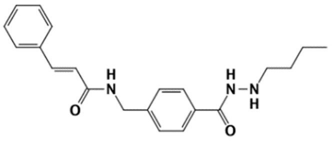 3c	training	8.44	[50]
22	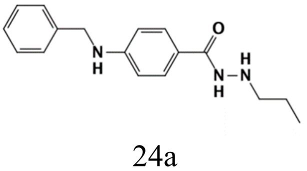 24a	training	8.21	[48]
23	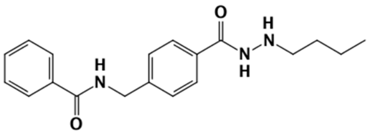 1l	training	8.07	[50]
24	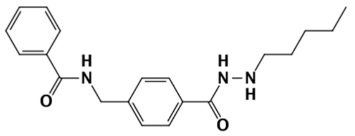 2e	training	7.73	[50]

25	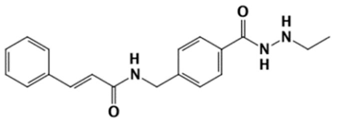 3a	training	7.71	[50]
26	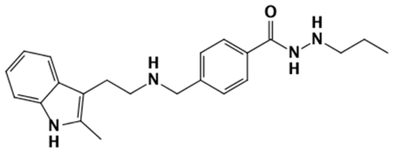 16a	training	7.70	[48]
27	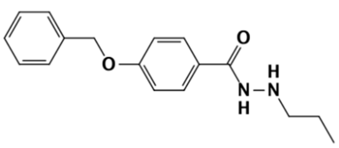 24c	training	7.61	[48]
28	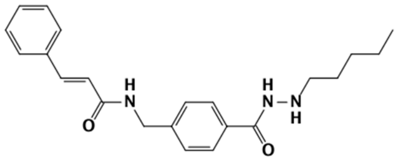 3d	training	7.49	[50]
29	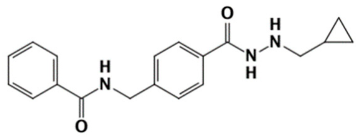 2g	training	7.40	[50]
30	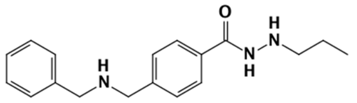 16c	training	7.01	[48]
31	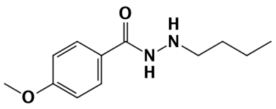 1a	training	6.80	[50]
32	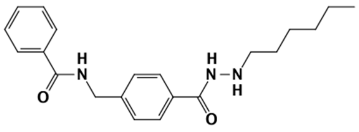 2j	training	6.73	[50]
33	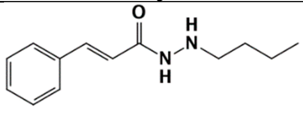 1i	training	6.53	[50]

34	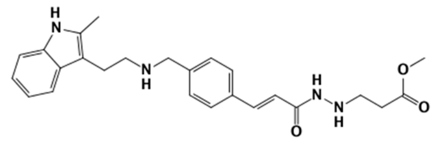 13d	training	6.51	[48]
35	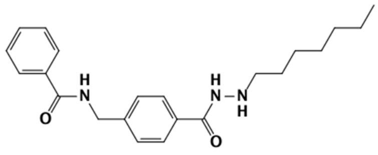 2h	training	6.25	[50]
36	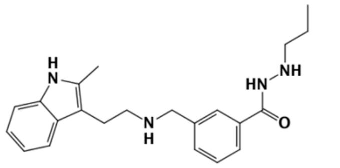 16b	training	6.17	[48]
37	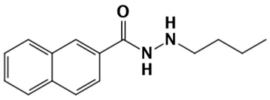 1k	training	6.05	[50]
38	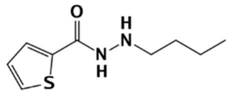 1f	training	6.04	[50]
39	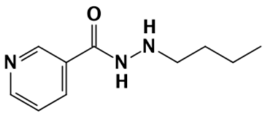 1d	training	5.81	[50]
40	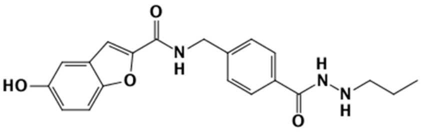 11m	test	9.29	[46]
41	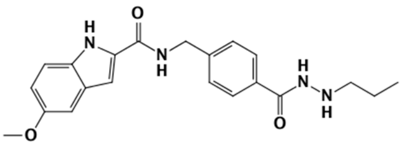 16d	test	9.24	[46]
42	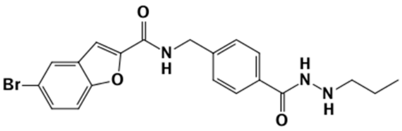 16g	test	9.21	[46]

43	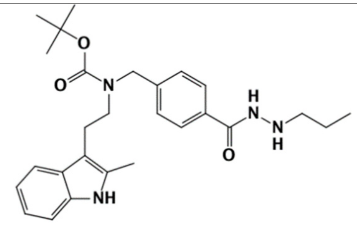<br>15a	test	7.90	[48]
44	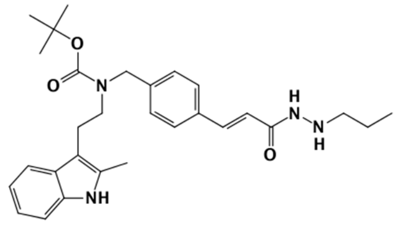<br>12a	test	7.78	[48]
45	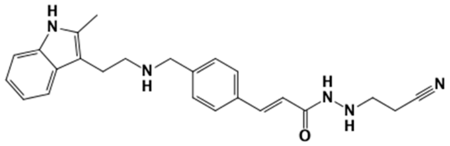<br>13c	test	7.46	[48]
46	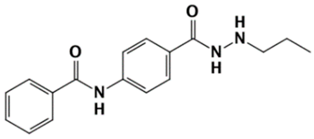<br>24b	test	7.31	[48]
47<br>(SR-3558)	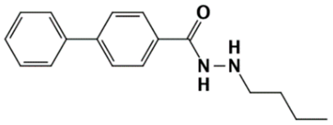<br>1e	test	7.17	[50]
48	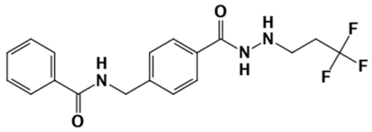<br>2f	test	7.16	[50]
49	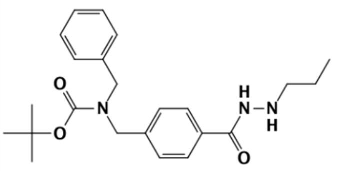<br>15c	test	7.11	[48]
50	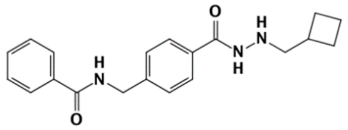<br>2m	test	7.07	[50]

51	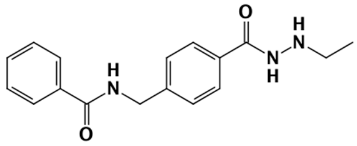 2l	test	6.73	[50]
52	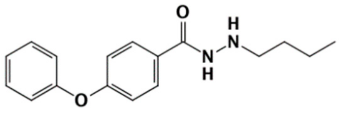 1c	test	6.51	[50]
53	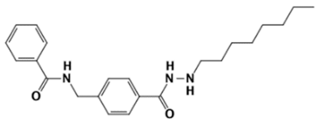 2i	test	5.96	[50]
54	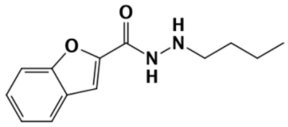 1b	test	5.87	[50]
55	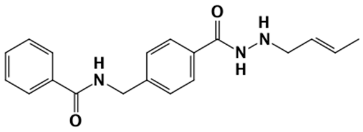 2a	test	5.81	[50]
56	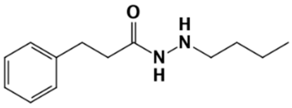 1h	test	5.72	[50]
57	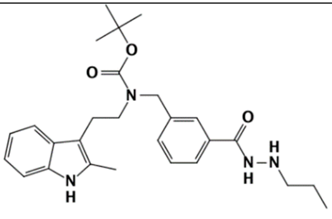 15b	In.	5.30	[48]
58	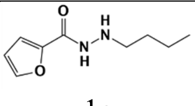 1g	In.	5.30	[50]
59	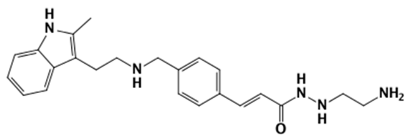 13b	In.	5.28	[48]

60	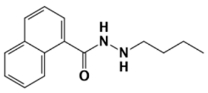 1j	In.	<5.00	[50]
61	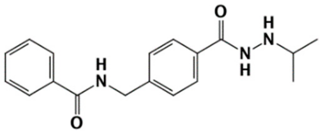 2b	In.	<5.00	[50]
62	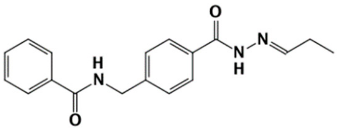 2c	In.	<5.00	[50]
63	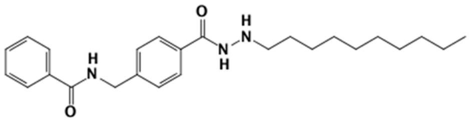 2k	In.	<5.00	[50]

Table S2. Prediction values of the best atom-based QSAR model generated for training set, test set and inactive set for HDAC3

Compound number	Compound name in original article	Class	HDAC3 experimental pIC_{50}	Prediction of atom-based-QSAR (pIC_{50})	Ref.
1	16h	training	9.55	8.69	[46]
2	13a	training	9.55	9.55	[48]
3	11i	training	9.42	9.07	[46]
4	11h	training	9.36	9.39	[46]
5	16f	training	9.21	9.11	[46]
6	16b	training	9.18	9.12	[46]
7	16e	training	9.08	9.11	[46]
8	11n	training	9.07	9.22	[46]
9	11d	training	9.05	9.02	[46]
10	3b	training	9.02	8.43	[50]
11	11e	training	8.99	8.88	[46]
12	11l	training	8.88	8.82	[46]
13	16c	training	8.88	9.42	[46]
14	11k	training	8.84	8.78	[46]
15	11c	training	8.83	8.91	[46]
16	11f	training	8.81	8.82	[46]
17	16a	training	8.79	9.09	[46]
18	11b	training	8.75	8.82	[46]
19	11g	training	8.69	8.93	[46]
20	2d	training	8.46	7.97	[50]
21	3c	training	8.44	7.78	[50]
22	24a	training	8.21	7.84	[48]

23	1l	training	8.07	8.18	[50]
24	2e	training	7.73	7.92	[50]
25	3a	training	7.71	7.85	[50]
26	16a	training	7.70	7.73	[48]
27	24c	training	7.61	7.08	[48]
28	3d	training	7.49	7.03	[50]
29	2g	training	7.40	7.31	[50]
30	16c	training	7.01	7.13	[48]
31	1a	training	6.80	6.46	[50]
32	2j	training	6.73	6.96	[50]
33	1i	training	6.53	6.67	[50]
34	13d	training	6.51	6.79	[48]
35	2h	training	6.25	6.40	[50]
36	16b	training	6.17	6.20	[48]
37	1k	training	6.05	5.97	[50]
38	1f	training	6.04	6.22	[50]
39	1d	training	5.81	6.03	[50]
40	11m	test	9.29	8.69	[46]
41	16d	test	9.24	8.87	[46]
42	16g	test	9.21	9.14	[46]
43	15a	test	7.90	8.43	[48]
44	12a	test	7.78	7.13	[48]
45	13c	test	7.46	7.17	[48]
46	24b	test	7.31	7.65	[48]
47	1e	test	7.17	6.92	[50]
48	2f	test	7.16	7.54	[50]
49	15c	test	7.11	7.96	[48]

50	2m	test	7.07	7.81	[50]
51	2l	test	6.73	7.88	[50]
52	1c	test	6.51	7.32	[50]
53	2i	test	5.96	7.85	[50]
54	1b	test	5.87	6.19	[50]
55	2a	test	5.81	8.08	[50]
56	1h	test	5.72	6.74	[50]
57	15b	inactive	5.30	7.54	[48]
58	1g	inactive	5.30	6.36	[50]
59	13b	inactive	5.28	7.56	[48]
60	1j	inactive	<5.00	6.11	[50]
61	2b	inactive	<5.00	7.96	[50]
62	2c	inactive	<5.00	7.97	[50]
63	2k	inactive	<5.00	7.19	[50]

Table S3. Prediction values of the best BFE model generated for training set, test set and inactive set for HDAC3

Compound number	Compound ID original article	Class	Docking score	HDAC3 experimental pIC_{50}	Prediction of BFE MODEL19_1 (pIC_{50})	Ref.
1	16h	training	-8.96	9.55	8.64	[46]
2	13a	training	-9.87	9.55	9.38	[48]
3	11i	training	-9.18	9.42	9.12	[46]
4	11h	training	-10.10	9.36	9.19	[46]
5	16f	training	-9.29	9.21	9.08	[46]
6	16b	training	-10.35	9.18	9.60	[46]
7	16e	training	-9.13	9.08	8.18	[46]
8	11n	training	-9.38	9.07	8.95	[46]
9	11d	training	-9.08	9.05	9.24	[46]
10	3b	training	-9.55	9.02	8.97	[50]
11	11e	training	-9.25	8.99	9.02	[46]
12	11l	training	-9.49	8.88	8.85	[46]
13	16c	training	-10.16	8.88	8.75	[46]
14	11k	training	-10.25	8.84	9.14	[46]
15	11c	training	-9.34	8.83	8.87	[46]
16	11f	training	-9.40	8.81	8.90	[46]
17	16a	training	-10.35	8.79	8.41	[46]
18	11b	training	-9.27	8.75	8.91	[46]
19	11g	training	-9.70	8.69	8.41	[46]
20	2d	training	-8.88	8.46	8.59	[50]
21	3c	training	-9.97	8.44	8.72	[50]
22	24a	training	-6.71	8.21	7.39	[48]
23	1l	training	-9.26	8.07	8.15	[50]

24	2e	training	-8.88	7.73	7.51	[50]
25	3a	training	-8.88	7.71	8.49	[50]
26	16a	training	-8.85	7.70	7.65	[48]
27	24c	training	-6.54	7.61	7.12	[48]
28	3d	training	-10.68	7.49	7.99	[50]
29	2g	training	-9.63	7.40	7.62	[50]
30	16c	training	-7.18	7.01	6.76	[48]
31	1a	training	-8.30	6.80	7.19	[50]
32	2j	training	-8.97	6.73	7.22	[50]
33	1i	training	-8.56	6.53	6.89	[50]
34	13d	training	-8.73	6.51	7.23	[48]
35	2h	training	-8.12	6.25	5.77	[50]
36	16b	training	-8.45	6.17	6.25	[48]
37	1k	training	-8.24	6.05	6.00	[50]
38	1f	training	-8.54	6.04	6.53	[50]
39	1d	training	-8.70	5.81	5.70	[50]
40	11m	test	-9.31	9.29	8.60	[46]
41	16d	test	-9.76	9.24	9.75	[46]
42	16g	test	-9.13	9.21	9.37	[46]
43	15a	test	-8.65	7.90	8.53	[48]
44	12a	test	-8.65	7.78	8.10	[48]
45	13c	test	-8.84	7.46	7.42	[48]
46	24b	test	-7.16	7.31	8.06	[48]
47	1e	test	-7.20	7.17	7.85	[50]
48	2f	test	-8.80	7.16	8.12	[50]
49	15c	test	-8.58	7.11	6.30	[48]
50	2m	test	-9.50	7.07	7.32	[50]

51	2l	test	-7.65	6.73	7.56	[50]
52	1c	test	-7.53	6.51	6.89	[50]
53	2i	test	-8.67	5.96	6.19	[50]
54	1b	test	-8.71	5.87	6.41	[50]
55	2a	test	-9.09	5.81	6.27	[50]
56	1h	test	-6.94	5.72	6.42	[50]
57	15b	inactive	-7.28	5.30	5.39	[48]
58	1g	inactive	-7.59	5.30	5.85	[50]
59	13b	inactive	-9.42	5.28	4.79	[48]
60	1j	inactive	-4.17	<5.00	4.43	[50]
61	2b	inactive	-9.60	<5.00	6.15	[50]
62	2c	inactive	-9.20	<5.00	5.48	[50]
63	2k	inactive	-7.49	<5.00	5.68	[50]

Table S4. 2D chemical structures and biological data of tested compounds.

Compound number	Molecular structure and ID in the original publication [53]	Class	HDAC3 experimental pIC_{50}	Ref.
64	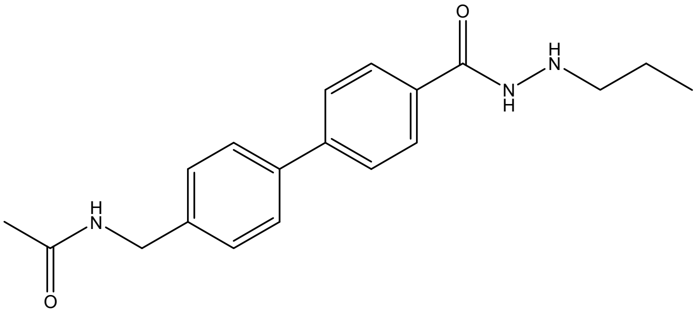 7a	Test	7.04	[53]
65	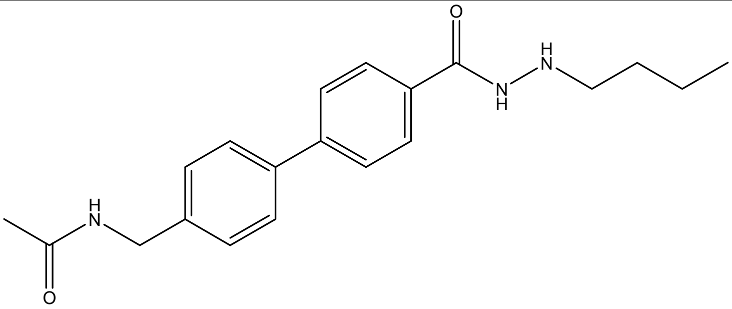 7b	Test	6.46	[53]
66	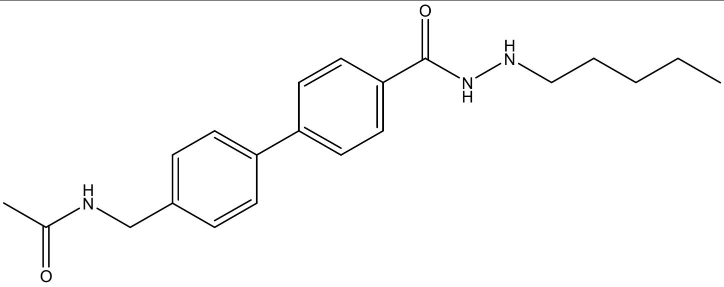 7c	Test	5.82	[53]
67	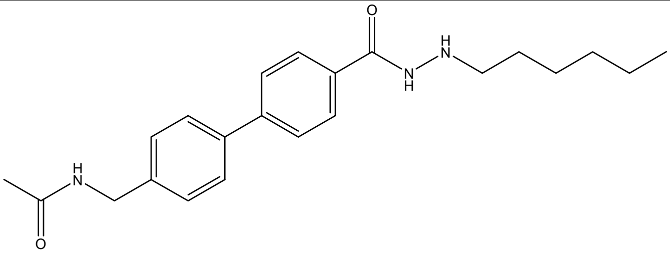 7d	Test	<5.00	[53]

68	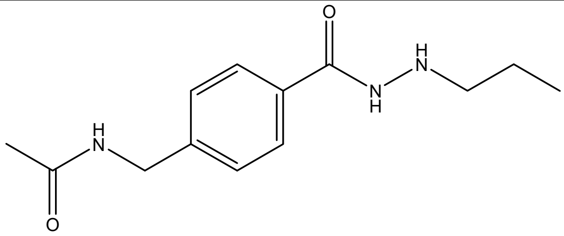 7e	Test	5.80	[53]
69	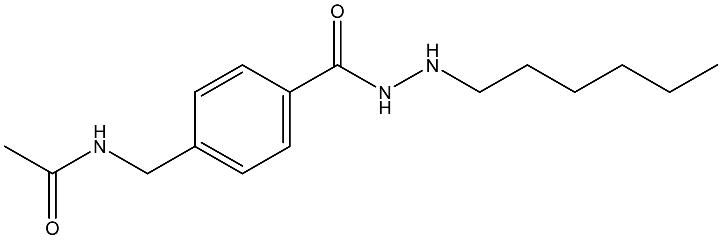 7f	Test	<5.00	[53]
70	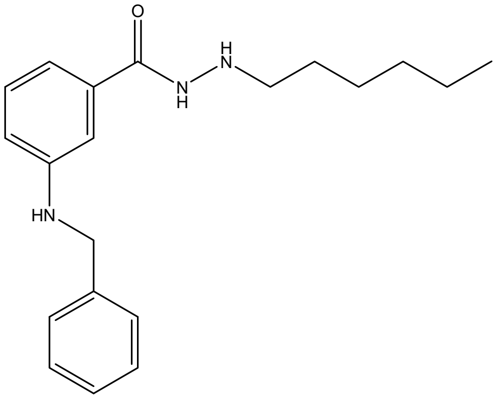 7g	Test	<5.00	[53]
71	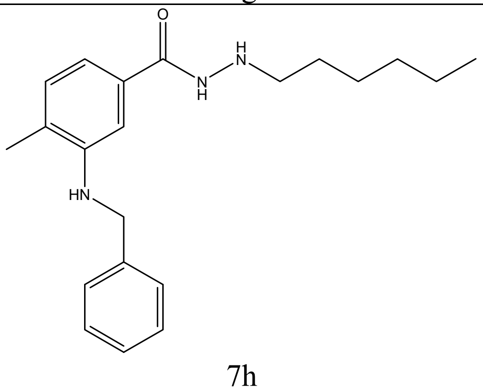 7h	Test	9%@1uM, 42%@10uM	[53]
72	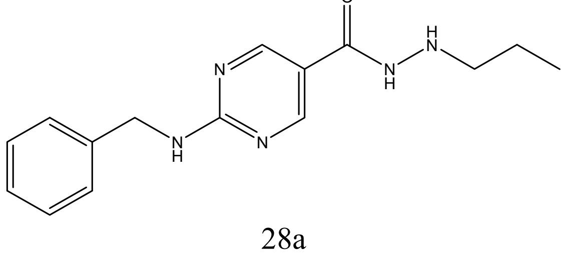 28a	Test	7.37	[53]

73	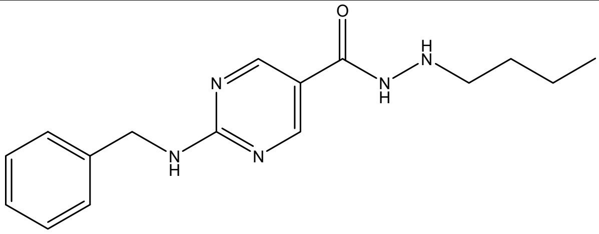<br>28b	Test	6.70	[53]
74	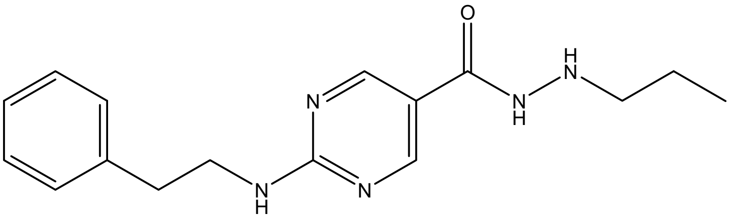<br>28c	Test	41%@1uM, 87%@10uM	[53]
75	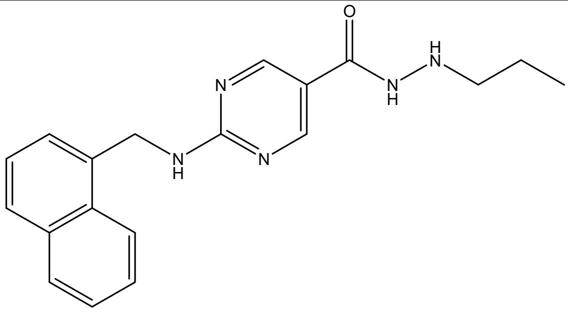<br>28d	Test	7.09	[53]
76	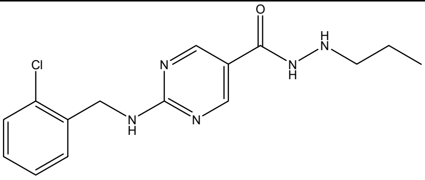<br>28e	Test	7.22	[53]
77	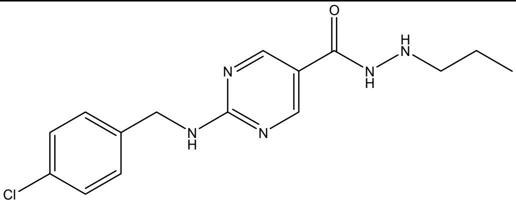<br>28f	Test	7.43	[53]
78	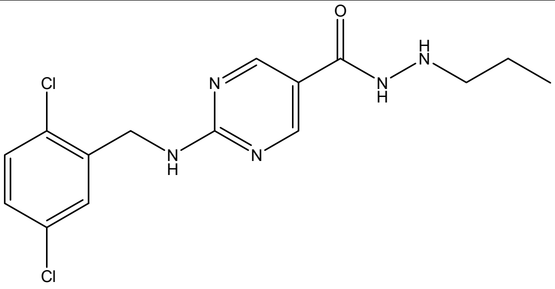<br>28g	Test	7.24	[53]

79	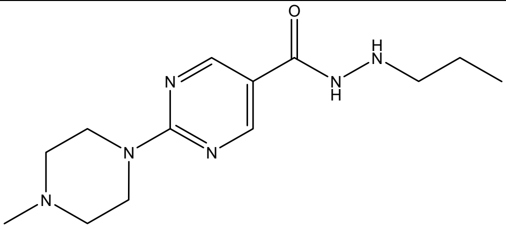 28h	Test	6.92	[53]
80	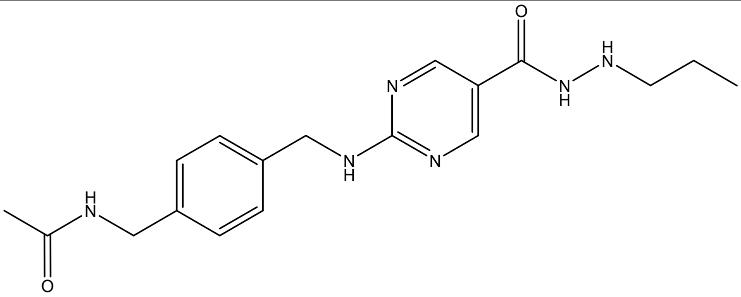 28i	Test	6.96	[53]
81	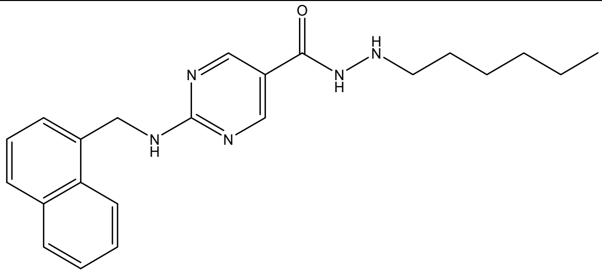 28j	Test	<5.00	[53]
82	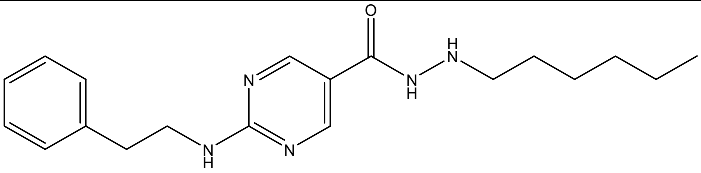 28k	Test	5.14	[53]
83	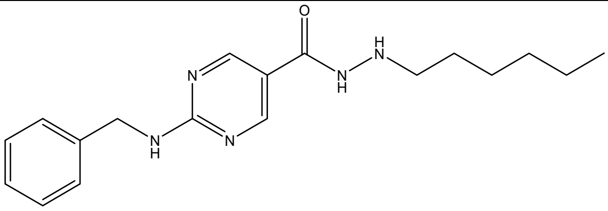 28l	Test	5.52	[53]
84	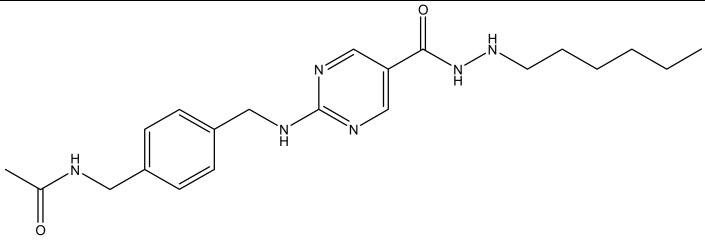 28m	Test	5.07	[53]

85	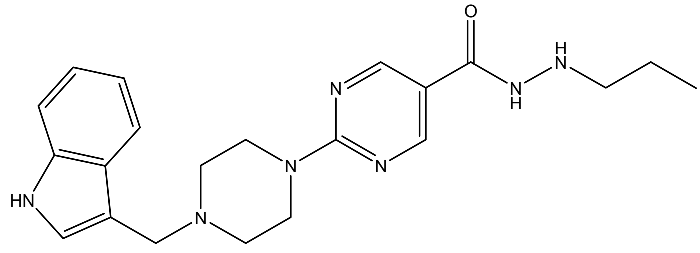 40a	Test	7.52	[53]
86	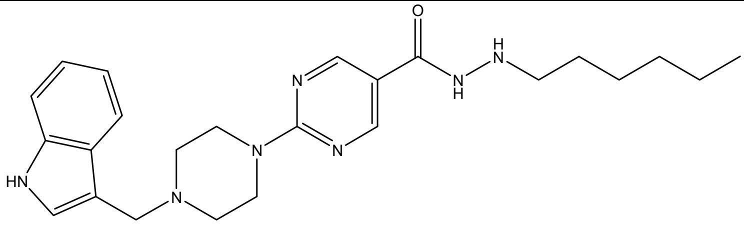 40b	Test	7.00	[53]
87	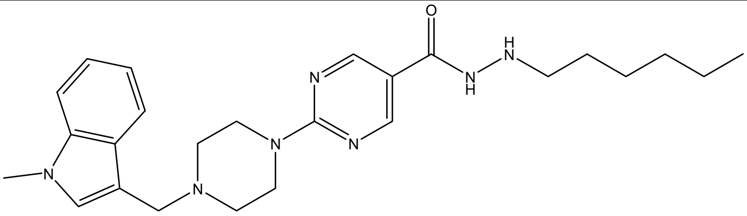 40c	Test	6.52	[53]
88	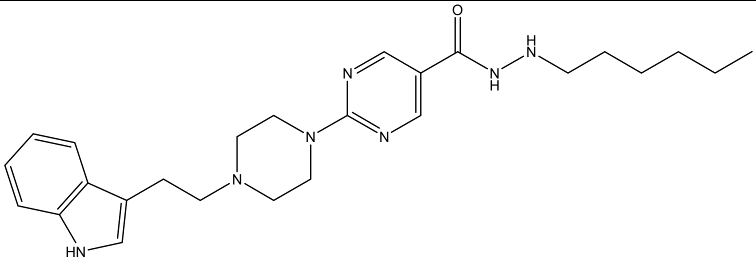 40d	Test	6.00	[53]
89	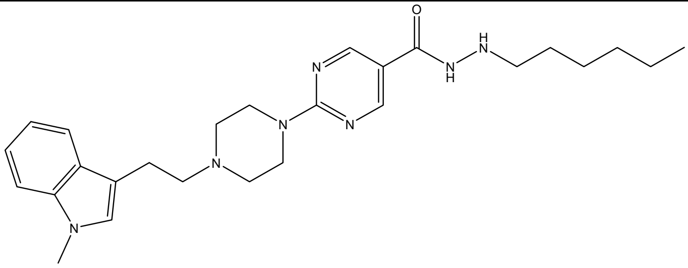 40e	Test	5.85	[53]

Table S5. Prediction values of the best atom-based QSAR model generated for training set, test set and inactive set for HDAC3

Compound number	Compound ID original article	Class	HDAC3 experimental pIC_{50}	Prediction of atom-based-QSAR (pIC_{50})	Ref.
64	7a	Test	7.04	7.03	[53]
65	7b	Test	6.46	6.59	[53]
66	7c	Test	5.82	6.57	[53]
67	7d	Test	<5.00	6.43	[53]
68	7e	Test	5.80	7.25	[53]
69	7f	Test	<5.00	7.05	[53]
70	7g	Test	<5.00	6.98	[53]
71	7h	Test	9%@1uM	6.82	[53]
72	28a	Test	7.37	7.85	[53]
73	28b	Test	6.70	7.29	[53]
74	28c	Test	41%@1uM	7.39	[53]
75	28d	Test	7.09	7.82	[53]
76	28e	Test	7.22	7.12	[53]
77	28f	Test	7.43	7.96	[53]
78	28g	Test	7.24	7.03	[53]
79	28h	Test	6.92	6.80	[53]
80	28i	Test	6.96	8.10	[53]
81	28j	Test	<5.00	6.54	[53]
82	28k	Test	5.14	6.86	[53]
83	28l	Test	5.52	7.25	[53]
84	28m	Test	5.07	7.61	[53]
85	40a	Test	7.52	7.63	[53]
86	40b	Test	7.00	7.18	[53]

87	40c	Test	6.52	6.52	[53]
88	40d	Test	6.00	7.40	[53]
89	40e	Test	5.85	7.13	[53]

Table S6. Prediction values of the best BFE model generated for tested compounds for HDAC3

Compound number	Compound ID original article	Class	Docking score	HDAC3 experimental pIC_{50}	Prediction of BFE MODEL19_1 (pIC_{50})	Ref.
64	7a	Test	-9.24	7.04	7.16	[53]
65	7b	Test	-9.48	6.46	6.89	[53]
66	7c	Test	-9.48	5.82	6.36	[53]
67	7d	Test	-7.33	<5.00	5.41	[53]
68	7e	Test	-6.87	5.80	6.69	[53]
69	7f	Test	-5.42	<5.00	5.07	[53]
70	7g	Test	-6.21	<5.00	3.92	[53]
71	7h	Test	-3.06	9%@1uM	0.24	[53]
72	28a	Test	-6.01	7.37	7.60	[53]
73	28b	Test	-6.35	6.70	6.68	[53]
74	28c	Test	-6.28	41%@1uM	7.29	[53]
75	28d	Test	-7.77	7.09	8.06	[53]
76	28e	Test	-8.13	7.22	6.86	[53]
77	28f	Test	-8.12	7.43	7.06	[53]
78	28g	Test	-7.90	7.24	7.98	[53]
79	28h	Test	-8.36	6.92	6.32	[53]
80	28i	Test	-8.09	6.96	7.42	[53]
81	28j	Test	-4.99	<5.00	1.65	[53]
82	28k	Test	-4.14	5.14	0.61	[53]
83	28l	Test	-5.41	5.52	3.26	[53]
84	28m	Test	-4.66	5.07	2.69	[53]
85	40a	Test	-7.80	7.52	7.64	[53]
86	40b	Test	-8.01	7.00	7.29	[53]

87	40c	Test	-7.59	6.52	6.86	[53]
88	40d	Test	-7.10	6.00	6.13	[53]
89	40e	Test	-7.76	5.85	6.11	[53]
